# Temporal dynamics of the fecal microbiome in female pigs from early life through estrus, parturition, and weaning of the first litter of piglets

**DOI:** 10.1186/s42523-024-00294-8

**Published:** 2024-02-21

**Authors:** Tara N. Gaire, H. Morgan Scott, Noelle R. Noyes, Aaron C. Ericsson, Michael D. Tokach, Hayden William, Mariana B. Menegat, Javier Vinasco, T. G. Nagaraja, Victoriya V. Volkova

**Affiliations:** 1 Department of Diagnostic Medicine/Pathobiology, College of Veterinary Medicine, Kansas State University, Manhattan, KS 66506 USA; 2 Department of Veterinary Pathobiology, School of Veterinary Medicine and Biomedical Sciences, Texas A&M University, College Station, TX 77843 USA; 3 Department of Veterinary Population Medicine, College of Veterinary Medicine, University of Minnesota, St. Paul, MN 55108 USA; 4 Department of Veterinary Pathobiology, College of Veterinary Medicine, University of Missouri, Columbia, MO 65211 USA; 5 Department of Animal Sciences and Industry, College of Agriculture, Kansas State University, Manhattan, KS 66506 USA

**Keywords:** Fecal microbiome, Aging, Physiological stages, Antimicrobial resistance, Female pigs

## Abstract

**Background:**

Age-associated changes in the gastrointestinal microbiome of young pigs have been robustly described; however, the temporal dynamics of the fecal microbiome of the female pig from early life to first parity are not well understood. Our objective was to describe microbiome and antimicrobial resistance dynamics of the fecal microbiome of breeding sows from early life through estrus, parturition and weaning of the first litter of piglets (i.e., from 3 to 53 weeks of age).

**Results:**

Our analysis revealed that fecal bacterial populations in developing gilts undergo changes consistent with major maturation milestones. As the pigs progressed towards first estrus, the fecal bacteriome shifted from *Rikenellaceae* RC9 gut group- and *UCG-002-*dominated enterotypes to *Treponema-* and *Clostridium *sensu stricto* 1*-dominated enterotypes. After first estrus, the fecal bacteriome stabilized, with minimal changes in enterotype transition and associated microbial diversity from estrus to parturition and subsequent weaning of first litter piglets. Unlike bacterial communities, fecal fungal communities exhibited low diversity with high inter- and intra-pig variability and an increased relative abundance of certain taxa at parturition, including *Candida* spp. Counts of resistant fecal bacteria also fluctuated over time, and were highest in early life and subsequently abated as the pigs progressed to adulthood.

**Conclusions:**

This study provides insights into how the fecal microbial community and antimicrobial resistance in female pigs change from three weeks of age throughout their first breeding lifetime. The fecal bacteriome enterotypes and diversity are found to be age-driven and established by the time of first estrus, with minimal changes observed during subsequent physiological stages, such as parturition and lactation, when compared to the earlier age-related shifts. The use of pigs as a model for humans is well-established, however, further studies are needed to understand how our results compare to the human microbiome dynamics. Our findings suggest that the fecal microbiome exhibited consistent changes across individual pigs and became more diverse with age, which is a beneficial characteristic for an animal model system.

**Supplementary Information:**

The online version contains supplementary material available at 10.1186/s42523-024-00294-8.

## Background

The mammalian maternal microbiome is recognized as a key determinant of overall host health and performance and is believed to contribute to the development of the offspring microbiome [1–3]. After birth, the gut microbiome undergoes a complex assembly process in both humans and animals [4, 5] and becomes more diverse with age. With the progression of age and transition from early life to adulthood, individuals undergo concurrent alterations in physiology, endocrinology, metabolism to support their developmental needs. These changes, occurring not only during early life to adulthood, but also subsequent gestation and pregnancy, also induce a significant shift in the compositions of the maternal gut and vaginal microbiomes in humans and animals [6–9]. The aging process imparts physiological effects on both the host and the microbiome [10]; thus, an ideal dataset for studying changes in the gut microbiome composition in females should be representative of early age through adulthood and onto parturition and weaning to fully encompass all key physiological stages of life.

Although several studies have characterized the composition of the pig fecal microbiome at specific or multiple stages of life, such as at an early age and/or from weaning to growing [11, 12] as well as during different stages of pregnancy and lactation [7, 13], the developmental trajectories of the fecal microbiome with age, from the early life period through first estrus, parturition, lactation and weaning, have not been well defined in the literature. In humans, dynamic relationships between age and microbial diversity in the gut are primarily visible in early childhood, young adulthood and during the different stages of pregnancy [14]. It is possible that fecal microbiome diversity and compositions are driven by host age [15], and in female pigs this may be stabilized by the time of first estrus or may continue to develop until parturition, emphasizing the need for longitudinal studies that examine the developmental trajectories of microbiomes starting from an early age and throughout the entire gestation period. Hence, understanding how the maternal microbiome changes with age and physiological stages (e.g., early life-estrus-parturition) may guide the development of targeted management strategies to improve maternal gut health and lactation performance.

The age-related alterations in the microbiome may directly or indirectly influence host health. Thus, the relationship between age and the gut microbiome is of particular interest due to the critical roles played by gut microbial communities in nutrient digestion, host health, and the host immune response [16, 17] as well as the composition of diverse microbes, many of which harbor resistance against several classes of antimicrobials. Furthermore, as the gut microbiome develops and matures throughout life, AMR gene compositions also change [18]. Additionally, the presence of pathogenic bacteria and their AMR determinants could pose additional risks to maternal health, and resistant bacteria could be transferred to offspring during parturition or during suckling [19]. As evidence of these risks, the presence of similar AMR gene profiles in the gastrointestinal tracts of mothers and their infants, and in the absence of direct antibiotic exposure, suggests that these resistance genes are readily and regularly passed from mother to infant [20, 21]. Furthermore, the transition to different physiological stages including estrus and gestation is a stressful event that can lead to an increased susceptibility to many opportunistic pathogens, including fungal species [22]. Nevertheless, the co-development of fungal communities during early life and the prenatal periods is not well understood. Most studies describing the human and pig gut microbiomes have focused mainly on the bacterial component; as a result, gut-associated fungal dynamics are poorly understood and remain largely unexplored both in humans and animals [23].

In humans, elucidating the dynamics of microbiome composition and AMR from the neonatal female through to pregnancy and subsequently lactation is extremely challenging; that is, any longitudinal study would span—at a minimum 4–5 federal research grants of 5 years each. Further, if such comprehensive data were available at the present time, it would include a cohort of individuals whose birth dates preceded most of the technological (i.e., DNA sequencing, bioinformatics) advances needed to analyze these very same microbiomes. Several factors, including antimicrobial exposure, diet, and environment, are known to influence both the microbiome and AMR dynamics; furthermore, these are exceedingly difficult to control in human studies. Thus, pigs are useful as a preferred monogastric animal model for studying the gut microbiome, especially given the similarities between pig and human anatomy, physiology, and metabolism as well as the similar developmental processes of the gut microbiome and their functions in the two species [24]. Additionally, nearly 96% of the functional pathways found in the human gut microbiome were also present in the pig gut microbiome, suggesting the suitability of pigs as models for human gut microbiota research [25].

In this study, we profiled the fecal microbiome, including both the bacterial and fungal community compositions, and AMR among fecal indicator bacteria from the early life period through first estrus, parturition and weaning in cohorts of female pigs (*n* = 18, 3–53 weeks of age) to test the hypothesis that fecal microbiome composition is largely a function of host age. Our analyses provide a broader understanding of the dynamics of the fecal microbiome and AMR from early life through fecund adulthood and may inform future microbiome investigations to improve health outcomes.

## Results

### Age-related progression of the fecal bacterial community from early life through estrus, parturition and weaning

**Fig. 1:**
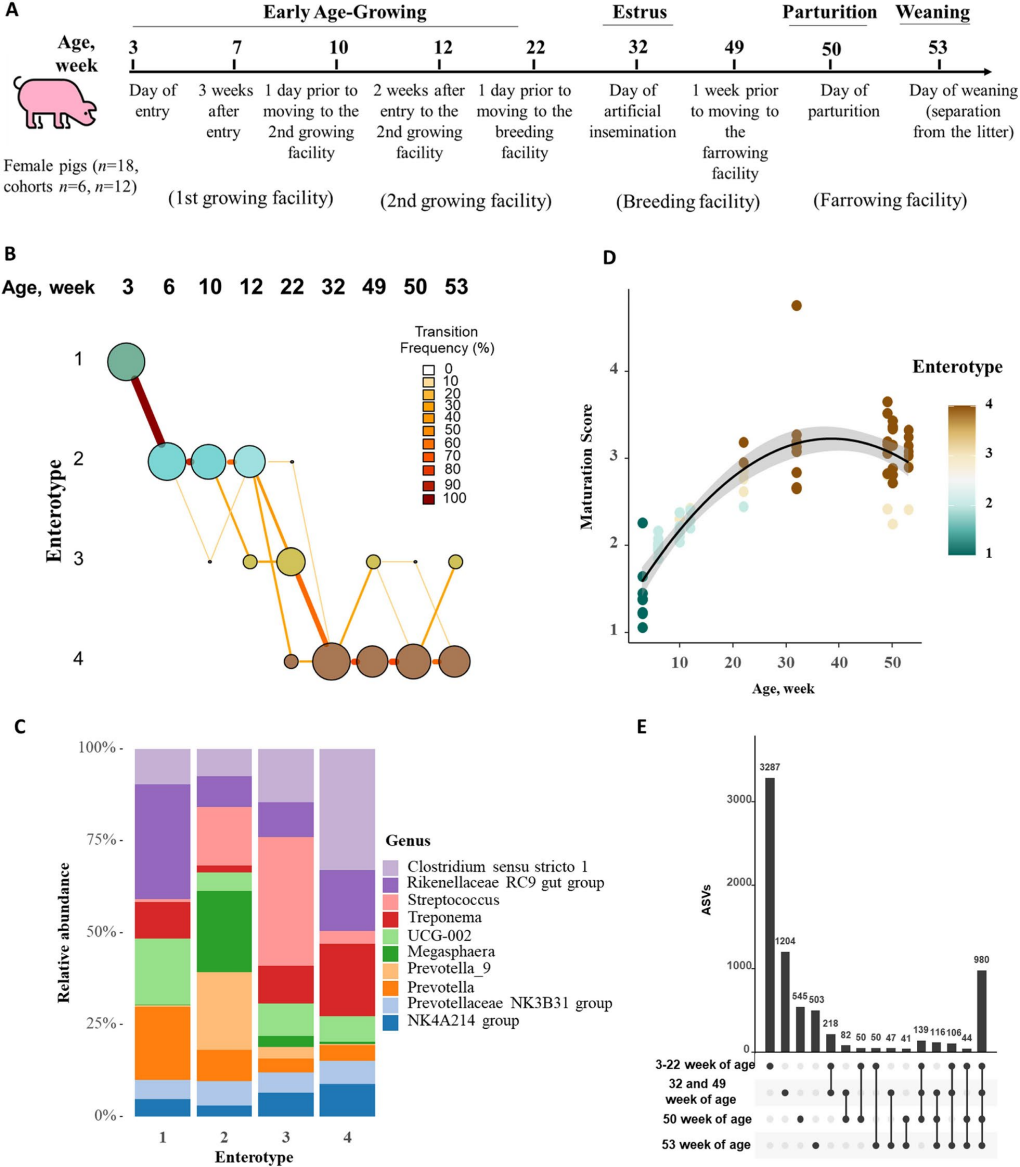
**A** Schematic representation of the study design. Two cohorts of female pigs (*n* = 18) were sampled at nine different age-points, from three weeks of age through estrus (i.e., at artificial insemination), a week before and at the day of parturition and weaning of the first litter. Fecal microbiome (*n* = 8 of 18 pigs per age-point) and phenotypic AMR (*n* = 18 pigs per age point) were characterized. **B** The fecal bacterial enterotype transition in female pigs from the earliest life period through estrus and parturition and weaning (first parity) (from 3 to 53 weeks of life). Enterotypes—representing specific community types within the fecal bacteriome. The entire 16S rRNA dataset (at the genus level) was classified into four distinct enterotypes based on the lowest Laplace approximation. **C** Stacked bar showing the mean relative abundance of the 10 most dominant bacterial genera in each enterotype. **D** Change in the bacterial maturation scores with age. The black line and shading show the mean with the 95% CI values. **E** Number of unique microbiome features (individual dots) shared (connected dots) and total (horizontal bars) ASVs among age points

Fecal samples were serially collected from eight female pigs (two cohorts, i.e., 3 weeks of age to estrus and/or artificial insemination, parturition, and weaning of first litter; at nine age points; for a total of 72 fecal samples, two cohorts) and subjected to microbiome profiling by sequencing of the V4 region of the 16S rRNA gene (Fig. 1A). A total of 10.9 million reads (average: 151.6 K, range: 0.8–184.2 K) was generated from the 72 samples. The mean raw read counts differed significantly by age/sampling point (*P* < 0.001), with samples collected at 3 weeks of age having significantly lower read counts than those collected at 10–53 weeks of age (all pairwise *P* < 0.01). However, the mean raw read counts did not differ when comparing the two cohorts (*P* = 0.47) and facility type (first and second growing, breeding, farrowing/parturition, Fig. 1) (*P* = 0.125). After filtering out low-quality reads using DADA2 [26], a total of 10.1 million reads remained across the samples; and after removing chimeric reads, a total of 7.3 million sequencing reads remained (Additional File 1: Table S1). To account for sequencing depth discrepancies across age points, samples were normalized using cumulative sum scaling (CSS) with default values [27]. A total of 7437 unique ASVs were identified across all age points and after removing 25 ASVs that were classified as not belonging to bacteria, 7412 ASVs were included in the downstream analysis.

Dirichlet Multinomial Mixture (DMM) models were fitted to the 16S rRNA gene sequencing data to identify enterotypes (i.e., community types) across the fecal samples. The models yielded four enterotypes (based on the lowest Laplace approximation) (Additional File 2: Fig. S1 A). Consistently across all pigs, we observed a strong temporal pattern of enterotype transition from 3 weeks of age to the time of first estrus and/or artificial insemination, followed by minimal changes until parturition and first litter weaning (Fig. 1B). The microbial communities of the pigs at 3 weeks of age were distinct from those at later ages, and the former all belonged to enterotype 1, which was characterized by a high proportion of the genera *Rikenellaceae* RC9 gut group, *Prevotella,* and *Lachnospiraceae UCG-002*, among others (Fig. 1C). As the pigs entered the growing stage and by 12 weeks of age, the fecal bacterial community underwent a profound shift from enterotype 1 to enterotype 2, and then transitioned to enterotype 3 at 22 weeks of age. Enterotype 2 was dominated by *Megasphaera*, *Streptococcus* and *Proviettaa_9,* while enterotype 3 was dominated by *Streptococcus*, *Clostridium *sensu stricto* 1* and *Treponema*. As the pigs progressed to estrus, the enterotype shifted to enterotype 4, dominated by *Treponema* and *Clostridium *sensu stricto* 1* (family *Clostridiaceae*). However, after estrus and until the end of lactation or the time of first litter weaning, most pigs’ microbiomes continued to be of enterotype 4, although some started to re-transition to enterotype 3 (Fig. 1B). Additionally, enterotypes were significantly (*envfit P* < 0.001) associated with bacterial alpha and beta diversity (Additional File 2, Fig. S1B-D), further demonstrating that the age-driven nature of fecal microbiome variations in the studied pig population.

To explore the potential relationship between fecal microbial community maturation and enterotypes, we estimated microbiome maturation scores following the method described by [28]. Notably, these scores exhibited a significant association with the transitions of enterotypes (Fig. 1D), highlighting the interconnectedness between microbiome development and shifts in enterotypes. Additionally, from 3 weeks of age to the growing stage (weeks 3–22), the pigs had more ASVs (i.e., ~ 3290). However, nearly 1200 ASVs were only identified during the first estrus and a week prior to parturition, while 545 ASVs were only detected during parturition (Fig. 1E). Only 139 ASVs were common across all of the growing, gestation and parturition stages, emphasizing the large amount of ecological turnover that occurs in the fecal microbiome of young female pigs.

**Fig. 2:**
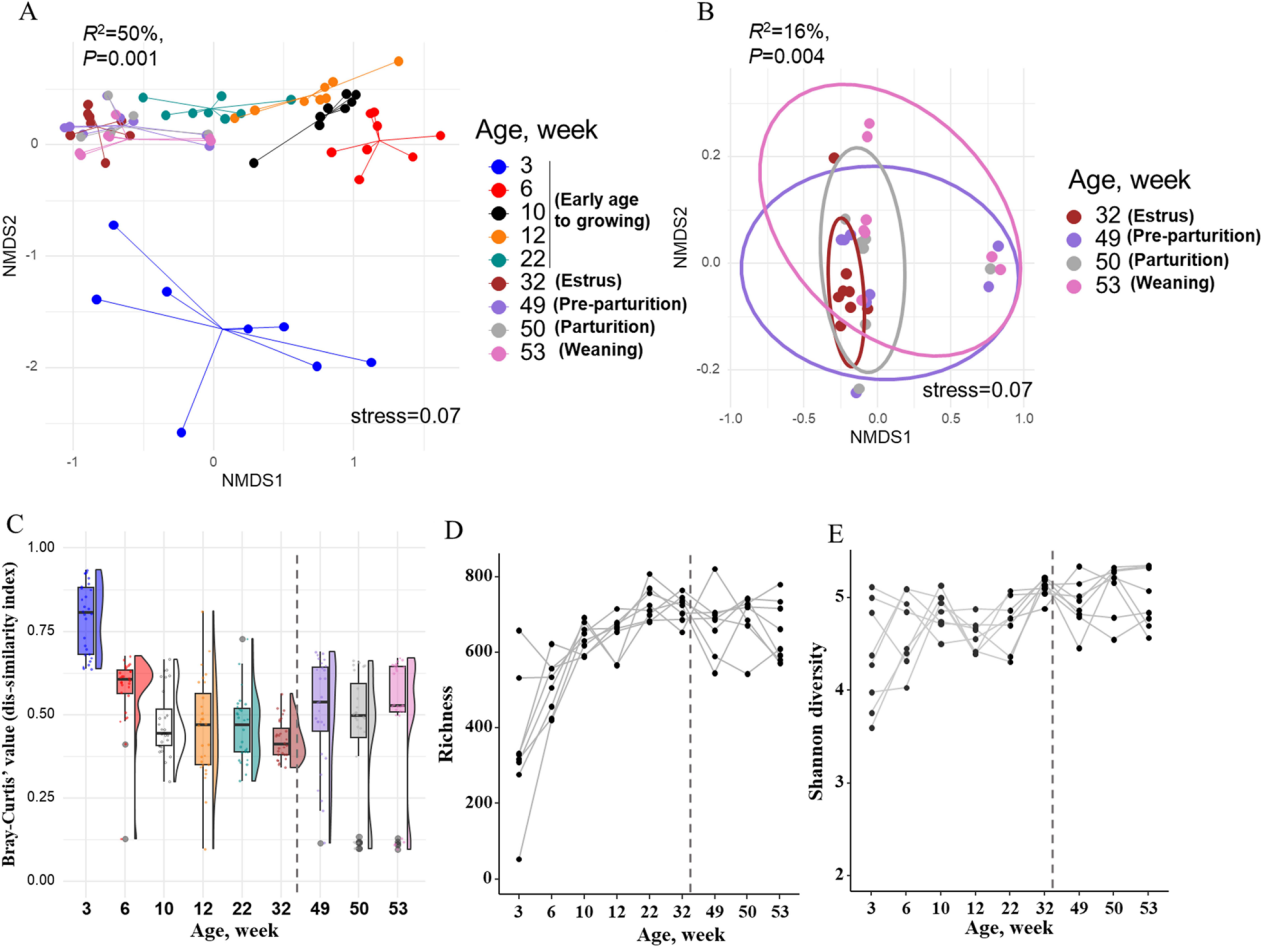
Overall fecal bacterial community composition in female pigs from the earliest life period to estrus (at time of artificial insemination), a week prior to parturition (pre-parturition) and the day of parturition and first litter weaning (3–53 weeks of age). **A** NMDS plot based on Bray‒Curtis dissimilarity between samples representing genus-level microbiome variation in pigs of all ages, and **B** during estrus and/or artificial insemination to parturition and weaning. Dots represent one sample and are colored by age, with the ellipse representing the 90% CI for data points from different physiological stages in female pigs (i.e., estrus and a week prior parturition, at parturition and at first litter weaning). The stress of the ordination, effect size (R^2^) calculated by multivariate permutational multivariate analysis of variance (PERMANOVA) tests and corresponding *P* values are shown in the plots. **C** Boxplots and distribution of the values of fecal bacterial community dissimilarity (Bray‒Curtis value) among ages. Bray‒Curtis values ranged from 0 to 1, with 0 being the least dissimilar (i.e., more similar) and 1 being the most dissimilar. **D**, **E** Line plots showing age-associated dynamics of fecal bacterial alpha diversity measured as the richness and Shannon diversity (at the ASV level) from 3 to 53 weeks of life

An NMDS plot based on Bray‒Curtis dissimilarity distances showed that the samples were clustered closely by age, with samples from the early age period to estrus and/or artificial insemination separated from those from parturition to weaning (age, R^2^ = 50%; estrus to weaning R^2^ = 16%, both *P* < 0.01) (Fig. 2A, B). This difference in the overall bacterial community composition was detected at nearly all taxonomic levels (R^2^ = 18–30%, *P* = 0.01; Additional File 3; Fig. S2 A–D). A higher Bray‒Curtis (BC) dissimilarity and beta-dispersion values from the bacterial community was detected at 3 weeks of age, but the composition became more similar during the growing phase at weeks 3–32 of age (Fig. 2C; Additional File 3; Fig. S2 E–H). However, as pigs progressed to parturition, their intra-timepoint variation started to increase again, indicating that the fecal bacterial composition of replacement female pigs near the end of gestation may diverge from that of the growing stage in some individuals (Fig. 2C). In a statistical model that considered both cohorts, the overall estimated Bray-Curtis dissimilarity values were higher during the period from early age to estrus (0.55, 95% CI 0.54–0.56) than during parturition and weaning (0.47, 95% CI 0.46–0.48).

In addition, the shift in enterotypes aligned with an overall increase in alpha diversity metrics (Fig. 2D, E) (Additional File 2, Fig. S1C, D). Number of observed ASVs (i.e., richness) and the Shannon diversity index both showed a significant increase over time, with samples at 3 weeks of age having the lowest number of observed ASVs. Furthermore, both metrices increased rapidly with age (LMM, *P* < 0.001 for both models) during the growing stage and were established at the time of first estrus and/or time of artificial insemination.

### Stability of the fecal bacterial community from estrus to parturition and weaning

Unlike the fluctuations in bacterial enterotypes and composition observed from 3 weeks of age to first estrus (week 32), the bacterial enterotypes and community composition both appeared to be relatively stable during the period from estrus through parturition to first litter weaning (i.e., end of lactation). Six pigs showed stable maintenance of enterotype 4, with 2 pigs transitioning back to enterotype 3. Furthermore, alpha diversity did not change significantly during this time period, and there was no distinct clustering of fecal samples collected at the end of lactation when compared to those collected at parturition (pairwise PERMANOVA, parturition *vs*. weaning, *P* = 0.438, Fig. 2B). Taken together, these results show that the fecal bacterial community structure changed profoundly from the earliest age period through the time of first estrus/and or artificial insemination, with minimal changes afterward, including through first parturition and weaning of the first litter.

### Bacterial genera associated with aging progression of fecal microbiome

**Fig. 3:**
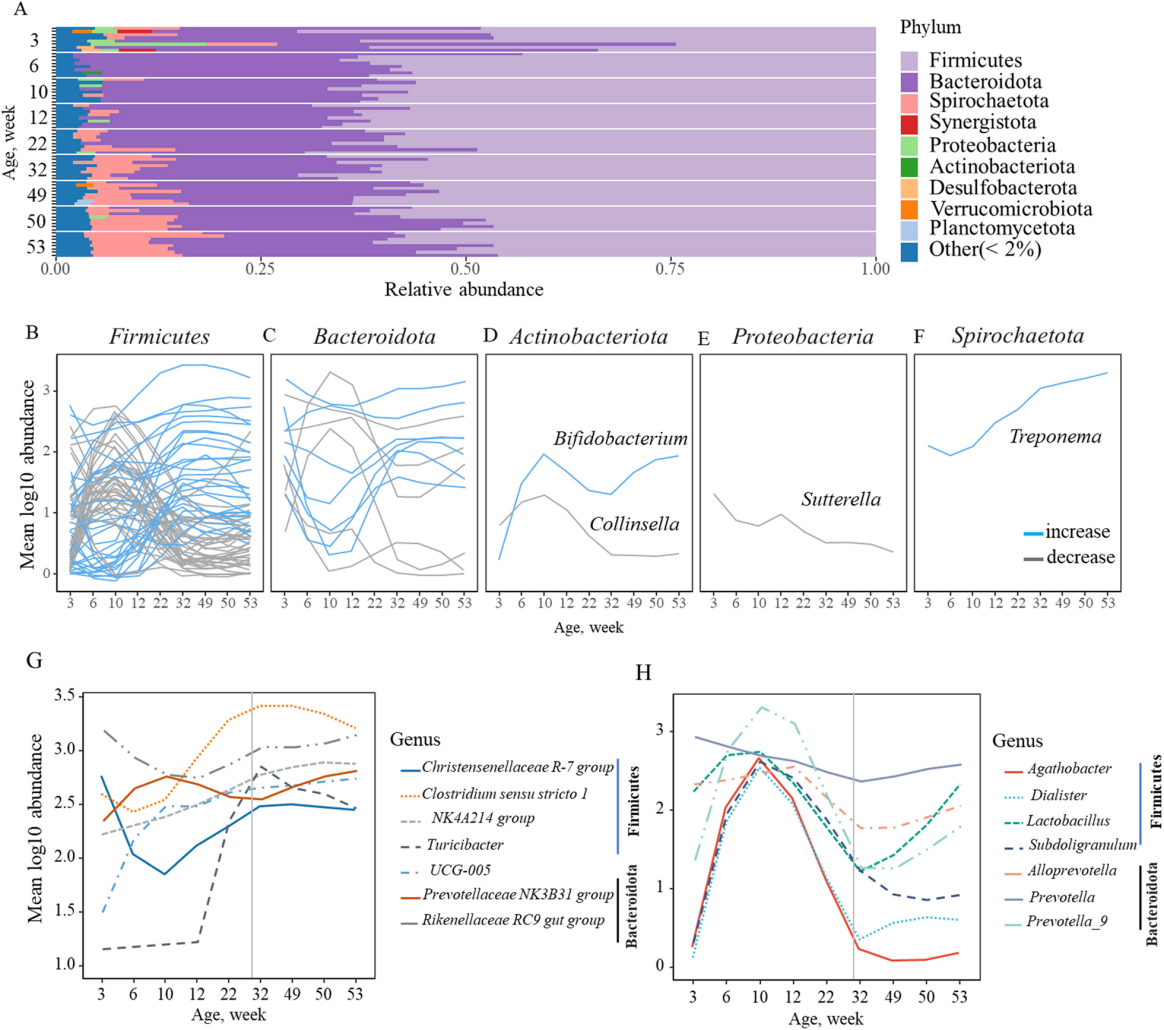
**A** Changes in the relative abundance of bacterial taxa (at phylum level) during the earliest life period through parturition and weaning of the first litter (3–53 weeks of age). **B**–**F** Mean relative abundance of bacterial genera, showing increases or decreases within each of the top 5 phyla (blue and gray lines represent individual genera whose abundances significantly increased or decreased over time). **G**, **H** Top seven genera collectively from the phyla *Firmicutes* and *Bacteroidetes*, with the highest relative abundance that either increased or decreased over time

The bacterial enterotype and composition analysis results suggested that the fecal bacterial community structure shifted across different age points. We then evaluated temporal trends in the relative abundance of specific bacterial taxa associated with age. Across all samples, the fecal microbiome was dominated by ASVs classified into the phyla *Firmicutes* (~ 58.4%) and *Bacteroidetes* (~ 32.6%), followed by *Actinobacteria* (~ 1%), *Proteobacteria* (1.5%) and *Spirochaetes* (4.5%) (Fig. 3A). Of these, the relative abundance of *Proteobacteria* decreased during the growing phase (week 3 to parturition, 4.5–0.7%, *P* = 0.02), while the relative abundance of *Spirochaetes* increased significantly during the 3 weeks of age to until weaning of the first litter (4.1–11%*, P* < 0.001).

Across all samples, *Clostridium *sensu stricto* (Clostridiaceae) 1*, *Streptococcus, Rikenellaceae* RC9 gut group, *Treponema, Butyricicoccaceae* UCG-002, and *Megasphaera* were the most abundant genera (Additional File 4: Fig. S3). We next explored the trajectories of relative abundance changes for bacterial genera from the earliest age period to parturition and first litter weaning by fitting multivariate time-series models on genus-level 16S RNA sequencing data using the maaslin2 R package. Using these results, we then grouped the bacterial taxa based on age-related abundance trajectories (i.e., significant increase or decrease, Benjamini‒Hochberg (BH) FDR adjusted *P* < 0.001). We found 44 and 47 genera whose relative abundance significantly increased or decreased, respectively, over time (BH FDR adjusted *P* < 0.01) (Additional File 5: Table S2). The relative abundance of several genera, such as the *Christensenellaceae* R-7 group, *Clostridium *sensu stricto 1, NK4A214 group, and *Turicibacter* UCG-005 from the phylum *Firmicutes* and the *Prevotellaceae* NK3B31 group and *Rikenellaceae* RC9 gut group from the phylum *Bacteroidetes*, increased significantly over time. Strikingly, *Bifidobacterium* and *Treponema* (from the phyla *Actinobacteria* and *Spirochaetes,* respectively) exhibited rapid increases during the gestation period (BH FDR *P* < 0. 01) (Fig. 3B–H, Additional File 5: Table S2). In contrast, genera in the phylum *Firmicutes*, including *Agathobacter*, *Dialister*, *Lactobacillus*, and *Subdoligranulum,* and genera in the phylum *Bacteroidetes*, including *Alloprevotella*, *Prevotella*, and *Prevotella_9,* decreased significantly over time (*P* < 0.05). A complete list of all differential abundance results is included in Additional File 5: Table S2).

### The fecal mycobiome does not exhibit the same clustering patterns as the bacteriome

**Fig. 4:**
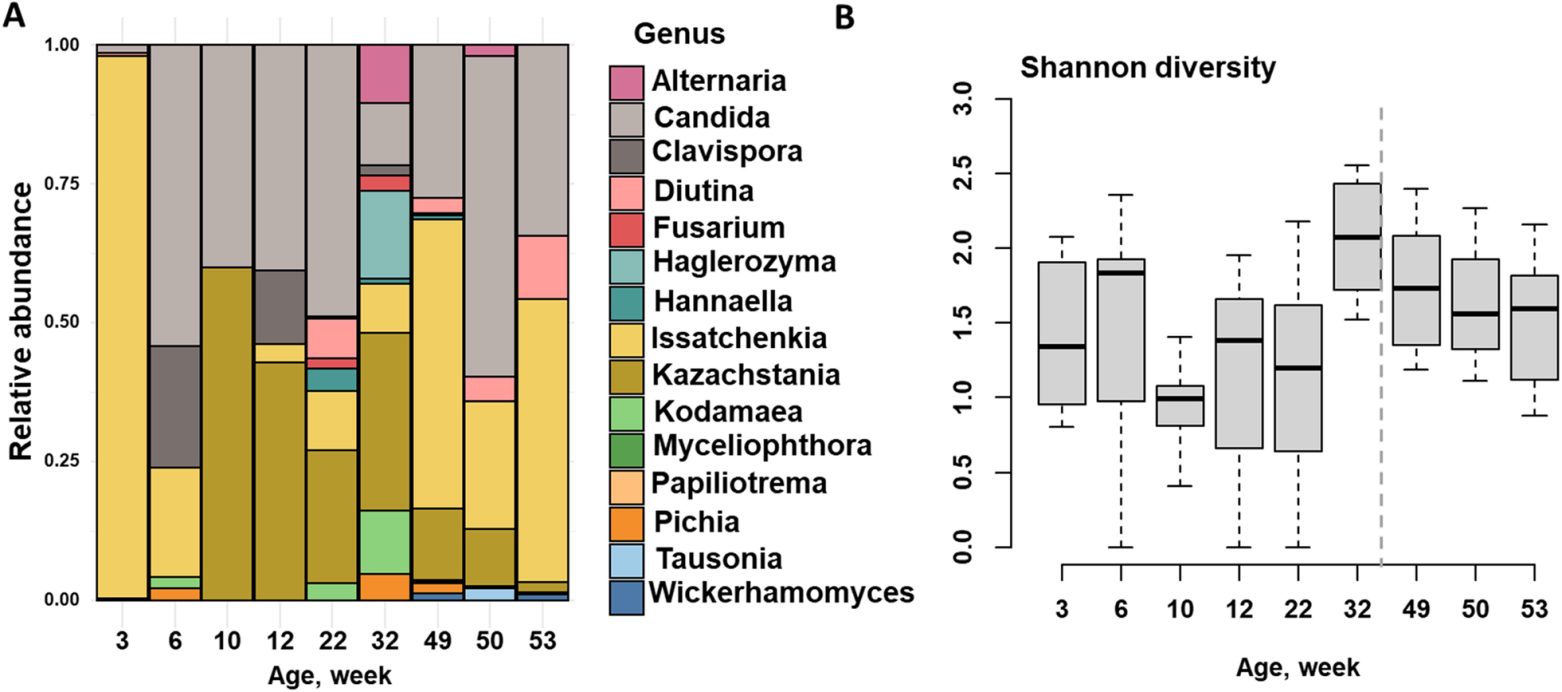
Fungal composition in female pigs from 3 to 53 weeks of life. **A** Relative abundance of fungi at the genus level. **B** Box plots showing age-associated dynamics of fecal fungal alpha diversity measured as the Shannon diversity from 3 to 53 weeks of life

While several studies have focused on the bacterial component of the fecal microbial community, other work has shown that the fungal component—the mycobiome—is also complex and multifaceted and influences host health [29]. In the present study, we found a total of 291 fungal ASVs, assigned to two fungal phyla, *Ascomycota* and *Basidiomycota,* with *Ascomycota* dominant (Additional File 6: Fig. S4 A The relative abundance of the fungal family *Saccharomycetaceae* was higher at three weeks of age compared to other ages; however, as the pigs progressed to parturition, the relative abundance of *Saccharomycetales_fam_Incertae_sedis* started to increase (Additional File 6: Fig. S4B).The relative abundance of *Issatchenkia*, *Kazachstania*, and *Clavispora*, among others, was higher at three weeks of age but subsequently declined with age, except for *Issatchenkia*, which exhibited an increase in relative abundance at the time of parturition (Fig. 4A). *Candida* spp. were detected at all age points, with a higher relative abundance observed at the time of parturition. In contrast to the bacterial composition, fungal diversity did not exhibit a consistent trend with age. However, overall diversity was highest during the time of estrus (Fig. 4B). Furthermore, the highest beta-diversity dispersion values were observed after 12 weeks of age until estrus (Additional File 6: Fig. S4 C).

### Phenotypic AMR

The dynamics of fecal antimicrobial resistance (AMR) in female pigs from an early age through farrowing and weaning of their first litter are largely uncharacterized, with most studies focusing on production pigs. Therefore, in order to understand the patterns of antimicrobial resistance in these pigs across various classes of antimicrobials, we subjected all samples from all 18 pigs for phenotypic AMR assessment. Overall, 72% and 50% of all samples (*n* = 162) carried coliforms and enterococci, respectively, that were resistant to at least one of the tested antimicrobials. A large proportion of these samples showed resistance to tetracycline (coliforms, 98%; enterococci, 90%). Overall, the counts of coliforms and enterococci with AMR (expressed as log_10_-transformed CFU/g feces) were highest at 3 weeks of age but exhibited substantial variation from the earliest age (week 3) through estrus and parturition and weaning of the first litter (week 53). Thus, we fitted hierarchical GAMs (“mgcv” package in R software) that allowed smooth trends to be estimated from the data using regression splines. The global GAM results indicated that there was a nonlinear relationship between the count of AMR coliforms or enterococci and pig age, with variations in the timing of significant trends across ages.

**Fig. 5:**
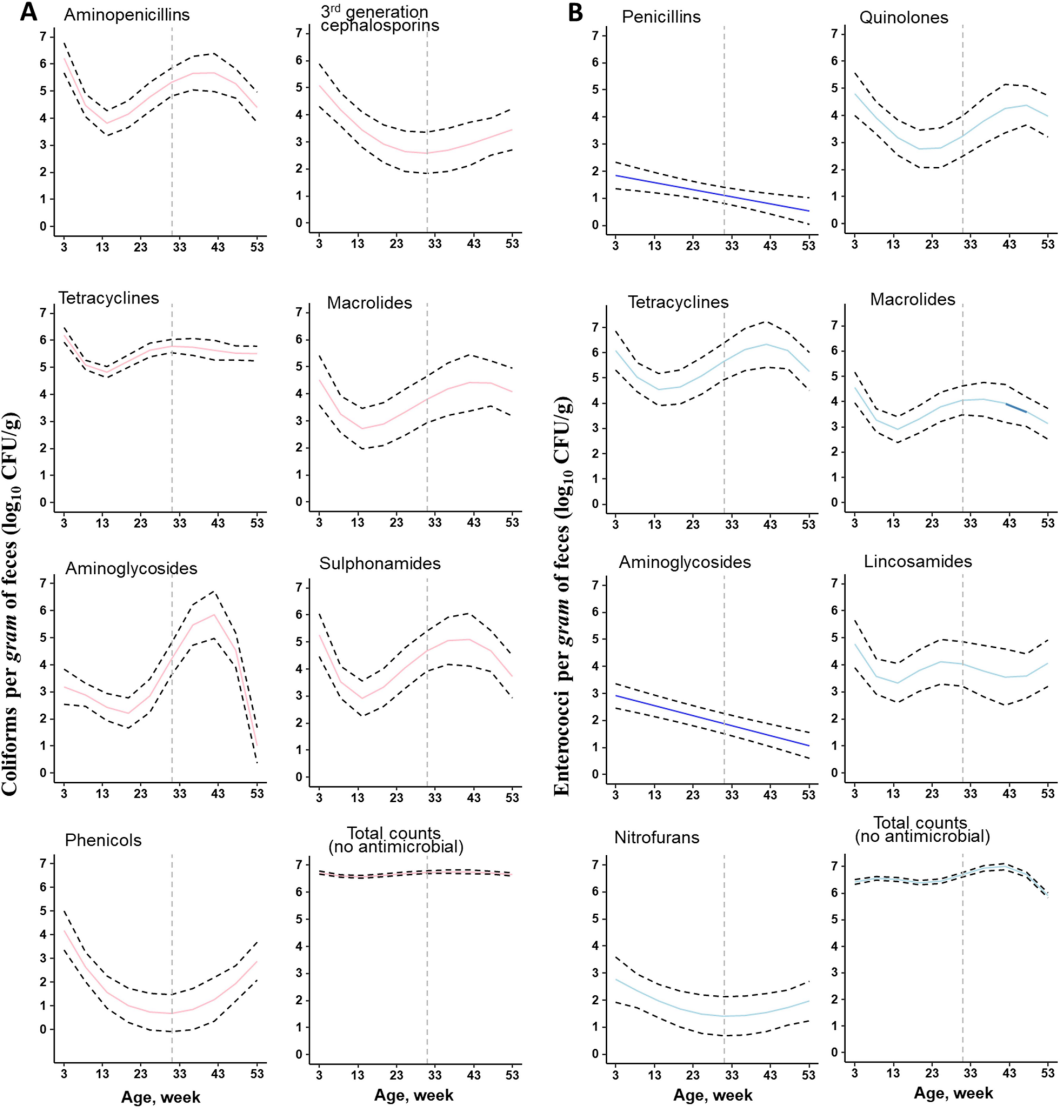
Counts (log_10_ CFU per g of wet feces) of fecal coliforms (**A**) and enterococci (**B**) in the presence of each antimicrobial by drug (i.e., bacterial counts growing in presence of the clinical breakpoint concentration of an antimicrobial of that class) and the total (without antimicrobial drugs) in relation to pig age. The dashed lines are the 95% CI on the spline-fitted age-dependent trend in the counts for each tested antimicrobial drug in relation to pig age

Overall, the counts of coliforms resistant to each of the tested antimicrobial classes showed significant declining trends of varying strength during the first 10 weeks of age (GAM, *P* < 0.01) (Fig. 5A). Interestingly, the counts of coliforms resistant to ceftriaxone, a third-generation cephalosporin, decreased consistently, and this trend persisted until 22 weeks of age (adjusted mean 4.8–2.4 CFU/g, GAM, *P* < 0.01). However, during the period between 12 and 32 weeks of age, we observed a significant increase in the counts of coliforms resistant to other antimicrobial drug classes, including aminopenicillins, tetracyclines, macrolides, aminoglycosides, and sulfonamides (GAM, all *P* < 0.01) (Fig. 5A). As the pigs progressed to gestation (between 32 and 50 weeks of age), the counts of coliforms resistant to ceftriaxone and phenicols showed increasing trends (GAM, *P* = 0.038, *P* = 0.009), while the counts of coliforms resistant to three other antimicrobial drug classes, namely, tetracyclines, aminoglycosides and sulfonamides, showed significant declining trends (*P* < 0.01) (Fig. 5A). From parturition to weaning of the first litter, which occurred at 50–53 weeks of age, only aminoglycoside-resistant coliforms showed significant trends, with declining counts. As expected, the total coliform count (growth without antimicrobials) was always higher than the counts of coliforms with AMR and remained similar throughout the study period (Fig. 5A).

Similarly, counts of enterococci resistant to each tested antimicrobial class, including quinolones, tetracyclines, macrolides, and lincosamides, also decreased during the first 8 weeks of age (GAM, *P* < 0.01) (Fig. 5B). The counts of enterococci resistant to nitrofurantoin showed decreasing trends during the first 10 weeks of age, but the trends were not significant. Strikingly, linear decreasing trends were observed for the counts of enterococci resistant to penicillin and aminoglycosides, which were significant throughout the study period (for both penicillin and aminoglycosides, GAM *P* < 0.01). During the period between 22 and 32 weeks of age, we observed significant increasing trends in the counts of enterococci resistant to quinolones, tetracyclines, macrolides and lincosamides, while nitrofuran-resistant enterococci showed a significant decline during the period from 12 to 22 weeks of age; however, there was some variation in the timing of the significant trends with age during this period. During the period from estrus to weaning of the first litter, only enterococci resistant to quinolones showed a significant increasing trend during the period from 32 to 49 weeks of age, while only enterococci resistant to macrolides showed a significant decrease during the farrowing stage (Fig. 5B). The counts of enterococci with AMR did not differ between the cohorts of pigs for each tested antimicrobial drug (GAM, *P* > 0.05). An apparent increase in total enterococci count (without antimicrobials) was observed until 32 weeks of age, and a significant decline was observed from 49 weeks of age to weaning of the first litter.

Given the decreasing trends in the counts of AMR coliform and enterococci with majority of tested antimicrobials in early life, we assessed the absolute and standardized (gene copies normalized to the total 16S rRNA gene copy number) abundance of two AMR genes, *tet*(A) and *bla*_CTX-M_, in wet feces as determined by qPCR. Interestingly, despite lower microbial diversity, the abundance of both AMR genes was higher at an early age but decreased afterward. Model-adjusted mean log_10_ copies per gram of feces for each of the two genes and standardized genes copy number (normalized to the total 16S rRNA gene copy number) and distribution of 16S rRNA gene copy number are presented in Additional File 7: Fig. S5. The mean log_10_ copy numbers of *tet*(A) (absolute and standardized) were high in the earliest life period (mean 7.10, 95% CI 6.76–7.45; − 3.6, 95% CI − 4.07 to − 3.13), with a rapid decrease observed during the first 10 weeks of age (*P* < 0.05), followed by a more modest increase during week 22 of age. However, the quantities of the genes remained similar from estrus and/or artificial insemination until weaning of the first litter (Additional File 7: Fig. S5) rising slightly during lactation. Similarly, the mean log_10_ copy numbers of *bla*_CTX-M_ (absolute and standardized) were also significantly associated with age (both *P* < 0.01). Higher absolute quantities of *bla*_CTX-M_ were observed at 3 weeks of age (mean 5.35, 95% CI 5.13–5.56), and then declined rapidly in the first 10 weeks of life. However, the abundance of genes started to increase during the period from week 10–22 of age while remaining relatively unchanged during the period from parturition to weaning of the first litter. In contrast, the 16S rRNA-standardized *bla*_CTX-M_ gene copy number was initially higher but declined in the first 6 weeks of the growing stage and then started to increase rapidly until week 32 of the growing stage (*P* < 0.02). During the parturition to weaning period, the quantities of genes remained relatively similar (Additional File 7: Fig. S5).

## Discussion

Age-associated changes in the composition and diversity of swine fecal microbiomes have been described, but most of this literature focuses on the early life period and/or commercial pigs being produced for entry into the food chain. We add to this body of knowledge by describing age-related fecal microbiome, fungal and AMR changes in piglets destined to become breeding stock; we contextualize these age-related changes in relation to important physiological stages including first estrus, parturition, lactation, and weaning of the first litter (i.e., 3–52 week of age). Relatively few longitudinal studies have been conducted to describe the dynamics of the gut microbiome in gravid hosts, including in both pigs and humans [7], with conflicting results reported in recent studies of pregnant women. The findings of one study [14] suggested that the microbiomes of various body sites (including fecal content) remained relatively stable, while the findings of a previous study showed a significant shift in the including fecal microbiome during the course of gestation [1]. In humans, performing longitudinal studies to profile microbial changes from a young age through gestation and pregnancy is challenging, and differences in overall results might be caused by many potential confounders, including environment and diet, which are almost impossible to control in human studies. In our study, female pigs were used as an animal model and were fed and housed similarly across all study subjects, providing insight into the dynamics of the fecal microbiome and AMR from the youngest age period through first estrus and weaning (end of lactation).

The fecal bacterial communities in female pigs exhibited an age-driven pattern, characterized by a rapid shift in composition and enterotypes from early life to the time of estrus (3–32 weeks of age), highlighting the crucial role of this period in microbiome development [30]. These findings are consistent with our previous studies, which demonstrated rapid assembly and increasing diversity of fecal microbial communities with age in adult pigs [18, 31]. In this study, the pigs were primarily fed a diet consisting of corn and soybean meal, with gradual adjustments made to the corn-to-soybean meal ratio to match their nutritional requirements as they grew. These dietary adjustments, however, were neither abrupt nor of significant magnitude. Despite changes in housing, diet, and production practices, the fecal microbiome composition showed similar dynamics of richness and diversity between early life and young age in both our current study of female breeding pigs and our previous cohort of production pigs [31]. Thus, the observed shifts in the microbial community and increased diversity do not seem to be solely attributable to changes in the diet and we observed an increase in microbial diversity and a decrease in AMR fecal bacteria with age, even in the absence of dietary changes. This suggests that age may be an important factor influencing the composition of the fecal microbiome in the studied pigs. Contrary to our hypothesis, the microbiome profile of females in this current study did not significantly change from estrus to lactation (32–72 weeks of age). The majority of pig fecal samples maintained the same enterotypes from parturition to weaning, with only a few transitioning to the *Streptococcus*-dominated enterotype. This stability was consistent across various taxonomic levels, with only modest changes in specific taxa during lactation. Similar fecal microbiome stability has been reported in lactating sows [13], possibly indicating functional similarities in the fecal microbiome during these stages. Further research is needed to elucidate the factors influencing the stability of the fecal microbiome during lactation, a critical period for sows and their piglets [32].

Age-related changes in the fecal microbiome have been associated with variations in AMR in fecal bacteria and gene quantities in pigs [18, 31]. When we performed phenotypic assessments of AMR within specific genera, we observed the highest counts of coliforms and enterococci with AMR; subsequently, these values also declined during the first 3 months and then varied greatly depending on the drug class. The highest resistance was detected against tetracyclines, and lower resistance was recorded against chloramphenicol. As the pigs progressed to estrus, increasing trends in the counts of coliforms resistant to the tested antimicrobial classes, including ceftriaxone, tetracyclines, macrolides, and phenicols, and the counts of enterococci resistant to the tested antimicrobial classes, namely, quinolones and tetracyclines, were observed.

Further, quantities of AMR genes (both absolute levels and the levels standardized to 16S rRNA gene copies) specifically, a tetracycline resistance gene (*tet*A) and 3rd-generation cephalosporin resistance gene (*bla*_CTXM_) copies within the bacterial community, were initially higher during the early life period, in the absence of antibiotic use and even when the fecal microbiome was least diverse. Later, all the values decreased during the first 2–2.5 months of age. Interestingly, the abundance of the *tet*(A) gene was elevated from 10 to 22 weeks of age/prior to breeding, but the *bla*_CTX-M_ gene did not exhibit this trend, which persisted until the time of estrus; however, as the pigs progressed to parturition, the levels all started to decline again. The presence of bacteria with AMR in the pigs fecal microbiome does not necessarily indicate that the individuals were exposed to those specific antimicrobials but could instead be attributed to the transfer of AMR genes through horizontal or vertical transmission between bacteria [33] and/or a shift in bacterial types to those harboring resistance genes. Thus, we suspect that the variation in the quantities among these stages could be attributed to age-related changes in the underlying bacterial population within the fecal microbiome [18, 34]. However, while we could not confidently link AMR gene-associated taxa, the consistent shift of microbial enterotypes from the early life to growing stages further suggests the possible association between underlying microbiome composition and associated fecal AMR dynamics.

Consistent with previous findings [33], the phyla *Firmicutes* and *Bacteroidetes* were most abundant in the pig gut across all age points, regardless of the physiological stage (i.e., early life to parturition and weaning). Consistent with the findings of a previous study [1, 13], relative abundance of *Actinobacteria* gradually increased during gestation. Notably, relative abundance of *Proteobacteria* did not increase as previously reported [1] but was instead similar to that observed by Liu et al. [13]. A striking observation in this study was that relative abundance of *Treponema* (phylum *Spirochaetota*), one of the dominant taxa in enterotype 4, increased rapidly during early age through parturition. Although a majority of the members of the *Treponema* genus were not classified at the species level, of those classified to species, we observed a higher relative abundance of *Treponema bryantii* as pigs progressed to estrus. Members of the genus *Treponema* include both commensal bacteria and pathogens [35], with *T. bryantii* recently linked to feed efficiency in sows [36] and reported to be present in the pig gut at the end of gestation [7]. A previous study found a significant interaction between host sex hormones and the spirochetes *Treponema* spp. [37], which was also associated with a specific period of the estrus cycle; however, further studies are needed to understand the specific roles of *Treponema* spp. during gestation. The relative abundance of *Bifidobacterium* (phylum *Actinobacteria*) also increased with age. *Bifidobacterium* is considered a beneficial bacterium and research has shown that the hormone progesterone can directly influence the composition of the gut microbiome in pregnant women, leading to an increase in the relative abundance of *Bifidobacterium* during late pregnancy [38]. Relative abundance of *Lactobacillus* spp. was depleted during the period from 3 weeks of age to estrus, but their relative abundance increased with the time of weaning of first litter. These findings suggests that certain bacterial genera within fecal microbiome may be associated with responses during different physiological stages, such as estrus and parturition in female pigs.

Although the bacterial constituents of gut microbial communities have been studied extensively, fecal-associated fungi and their roles are poorly understood in humans [23] as well as in pigs [39]. In our study, fewer fecal-associated fungi were detected across all age points, and their diversity did not follow the same age-related pattern as the fecal bacterial community (Fig. 5). In our study, *Ascomycota* and *Basidiomycota* are two dominant phyla and *Candida*, *Kazachstania*, *Issatchenkia*, and *Diutina* are the most abundant genera across all samples. These fungal taxa also reported on the gut mycobiome of the Human Microbiome Project healthy cohort (HMP) [40, 41]. Interestingly, we noticed a higher abundance of fungal genera, including *Candida* and *Kawachstania* spp., at parturition. Our study revealed that approximately 20% of fungal ASVs were classified as known fungal genera, and previous work from the HMP mycobiome study [41] reported a similarly lacking taxonomic information. ITS sequencing provides greater resolution of fungal constituents of the microbiome; however, the lower numbers of fungi in the GI tract and several technical challenges, including the sparse database and accuracy of taxonomic information, present challenges to identifying key fungal features. This suggests standardizing fungal databases, DNA isolation and sequencing methods, and bioinformatics data analysis will be crucial for comprehensive mycobiome data analysis in the future.

In our study, we observed that physiological age was associated with fecal microbial changes in growing female pigs. However, it is important to acknowledge that the small sample size (8 pigs) of our fecal microbiome study may limit the generalization of our findings to a broader population of pigs. Further, it is crucial to highlight that the pig fecal microbiome has been shown to be influenced by a multitude of factors, including gastrointestinal anatomical sites and the introduction of solid feed, which have been identified as influential determinants shaping bacterial communities in commercial pigs from farrow to finish [42]. Host genetics may also play a significant role in gut microbiome differences, as demonstrated in studies on the influence of breed on microbiome variations [43, 44]. Notably, during the early stages of piglet life, the influence of nursing mothers and breed was apparent, but it was the introduction of solid feed and subsequent weaning that predominantly drove the succession of the gut microbiome [45]. Moreover, diet, age, body weight, and antimicrobial exposure have been established as significant factors shaping the gut microbiome of productions pigs [30, 46, 47]. Additionally, a different investigation emphasized the substantial impact of weaning on the piglet gut microbiome, revealing significant alterations in both bacterial and fungal communities post-weaning [48]. These collective findings underscore the complexity of factors contributing to microbial changes in pig gut ecosystems, emphasizing the need to consider multiple variables when studying fecal microbiome dynamics in swine.

## Strengths and limitations

Despite the increase in microbiome research, there is still insufficient published research on microbiome dynamics and AMR, particularly from the early life period through estrus and lactation/weaning in females. The major strengths of this study include the following: (a) its longitudinal design; importantly, we followed animals from the 3 weeks of age through estrus, parturition and weaning (3–53 weeks of age, i.e., parity 1) to capture the trajectories of the fecal microbiome and mycobiome and during the entire period of first parity, using sequence-based techniques (16S rRNA gene and ITS based); and (b) the quantification of AMR fecal bacteria within the bacterial community using a culture-based approach that provides broader insight into whether the antimicrobial-resistant fecal bacterial population changes from the early life period through parturition and weaning.

Although 16S rRNA sequencing is widely used to characterize the microbial community across different sample types, this approach also has limitations, including the inability to classify all sequence features at the species level [49]. For instance, in our study of 142 ASVs that were classified as *Treponema*, only four (and none of the *Bifidobacterium* ASVs) were classified at the species level. Thus, future studies using multiple approaches, including species-level identification (e.g., with shotgun sequencing), will provide additional insights into bacterial species and associated AMR genes, supplementing those gained from this study. In addition, applying absolute quantification of bacterial or fungal taxa (e.g., measuring 16S rRNA gene copies by qPCR or using ITS sequencing) would be more relevant for describing the actual abundance of taxa with age. Similarly, changes in the maternal microbiome occur beyond the gut; thus, microbiomes at other body sites, including vaginal microbiomes [50], along with the hormonal profile should be considered for future studies, and such studies will undoubtedly shed light on how microbes interact with each other and their association with health. We acknowledge the limitations of performing microbial culture-based phenotypic AMR analysis on just two indicator bacteria that may not represent the true source population (i.e., the fecal microbial community); however, these two broad categories of gram-negative and gram-positive bacteria are representative of clinically important pathogens, and their resistance profiles are highly relevant.

## Conclusions

In conclusion, this study highlights the temporal dynamics of the fecal microbiome and AMR bacteria from the early life period to estrus, parturition and weaning in female pigs. The pig fecal microbial composition was substantially shifted from the 3 weeks of age to estrus, but limited changes occurred after estrus until weaning. Unlike the pig fecal bacterial communities, the pig fecal fungal taxa had low diversity and did not appear to undergo a similar age-pattern to those of the bacterial community. Changes in the fecal microbial community were associated with the count of fecal bacteria with AMR and of quantities of AMR genes—specifically, a tetracycline resistance gene (*tet*A) and 3rd-generation cephalosporin resistance gene (*bla*_CTXM_) copies—within the bacterial community, were highest in the youngest pigs but subsequently changed with age as they progressed to adulthood. These findings may also contribute to the understanding of human fecal microbiome dynamics, given the similarities between the gastrointestinal (GI) tracts of pigs and humans.

## Materials and methods

### Study design, animals, and sample collection

This study was performed at Kansas State University Segregated Early Weaning Facility (IACUC #3528) and Swine Research and Teaching Unit (IACUC #3529). A total of 18 female weaned piglets (two cohorts of *n* = 6 and *n* = 12) were randomly selected upon arrival at the farm. The piglets were a commercial crossbreed with a Landrace base for one line and a Large White base for the other (DNA 200 × 400, DNA Genetics, Columbus, NE). The pigs were initially housed at the Early Weaning Facility until they reached 10 weeks of age and were later moved to the Swine Teaching and Research Unit. They were followed from 3 weeks of age through estrus, artificial insemination, parturition (farrowing), and the weaning of their first litter of piglets.

Pigs were vaccinated with the Porcine circovirus Type 2 vaccine (Circumvent PCV, Merck Animal Health, Rahway, NJ; 2 mL intramuscular[IM]) at 2 and 5 weeks of age and again 3 weeks prior to breeding. They also received vaccinations for Leptospira and Parvovirus at 5 and 3 weeks prior to breeding (Farrowsure Zoetis Animals Health, Kalamazoo, MI; 2 mL IM). Additionally, they were vaccinated for *Clostridium perfringens* Type C and *E. coli*, along with a *Haemophilus parasuis* bacterin (Littergard, Zoetis Animal Health; 2 mL IM), at 5 and 3 weeks before farrowing. In each facility, the pigs were fed mainly corn‒soybean meal grain-based diets given based on the growing phase as per National Research Council (NRC, 2012). The nursery diets contained zinc oxide (3000 ppm from day 0 to 10 and 2000 ppm from day 10 to 21) and no other antimicrobials or antibiotics administered in the feed, water or by injection for the remainder of the study. Study pigs were housed in dedicated pens and were not housed with other pigs in their pens, with ad libitum feeders from the time of entry to the farm until estrus detection. After being found in estrus, they were housed and fed approximately 2.7 kg per day in individual pens until approximately day 40 of gestation. From day 40 of gestation until parturition (farrowing), the pigs were housed in a group pen with electronic feeders and were fed approximately 2 kg per day. The pigs were moved to the farrowing facility approximately 7 days prior to parturition and fed with electronic feeders. During lactation, the pigs were fed ad libitum.

A fecal sample was collected from each animal *per rectum* at each of the following nine age points: 3 weeks (at entry into the first growing facility), 6 weeks (21 days after entry into the first growing facility), 10 weeks (a day before being moved to a second growing facility), 12 weeks (14 days after being moved to the second growing facility), 22 weeks (a day prior to being moved to the breeding facility), 32 weeks (at estrus and/or artificial insemination), 49 weeks (7 days prior to being moved to the farrowing (parturition) facility), 50 weeks (on the day of parturition) and 53 weeks (on the day of weaning of the first litter and/or end of lactation) (Fig. 1A). A total of 162 fecal samples were collected longitudinally from the 18 pigs. The fecal samples were kept on ice after each sampling, and both whole feces and fecal aliquots mixed with 50% glycerol were stored at − 80 °C until DNA extraction and quantification of total and AMR coliforms and enterococci, respectively.

### DNA extraction and 16S rRNA gene and internal transcribed spacer (ITS) sequencing

A total of eight pigs randomly selected from the 18 pigs (4 from each cohort) and a total of seventy-two of their fecal samples (8 pigs × 9 age points) were subjected to DNA extraction for 16S rRNA gene sequencing.

Briefly**,** whole fecal samples were thawed and homogenized by vortexing, and genomic DNA was extracted from each fecal sample using the protocol published by [51] with the modification recommended by Korte and his colleagues [52] for 16S rRNA gene sequencing. The DNA concentration was measured via fluorometry (Qubit 2.0, Life Technologies, Carlsbad, CA) using Quant-iT broad-range (or high-sensitivity) dsDNA reagent kits (Invitrogen, Carlsbad, CA). For sequencing of the V4 hypervariable region of the 16S rRNA gene, 100 ng of metagenomic DNA was amplified using universal primers (U515F/806R) [53, 54] with the following protocol: 98 °C (3 min) + [98 °C (15 s) + 50 °C (30 s) + 72 °C (30 s)] × 25 cycles + 72 °C (7 min).

After amplification of the V4 hypervariable region of the 16S rRNA gene, the amplicon pool was combined, mixed, and purified by adding Axygen AxyPrep Mag PCR Clean-Up Beads (Axygen Corning Life Sciences, Glendale, AZ, USA) (50 µL of beads was thoroughly mixed with 50 µL of amplicons). The pooled amplicons were quantified with the Quant-iT HS dsDNA Kit on a fragment analyzer (Advanced Analytical, IA, USA). Similarly, to identify gut fungal community, fungal ITS DNA amplicons were constructed by amplification of the ITS region of nuclear DNA with primers flanked by Illumina standard adapter sequences. Briefly, universal primers (ITS1-30F/ITS1-217R) were used to amplify the ITS 1 region. Extracted genomic DNA was quantified by Qubit flourometer using the quant-iT HS dsDNA reagent kit (Invitrogen). PCR reactions (50 ul) contained 100 ng of genomic DNA, forward and reverse primers (0.2 μM each), dNTPs (200 μM each), and Phusion High-Fidelity DNA Polymerase (1 U). Amplified product (10 μl) from each PCR reaction was combined and thoroughly mixed to make a single pool. Pooled amplicons were purified by addition of Axygen AxyPrep MagPCR Clean-up beads to an equal volume of amplicons. Purification followed the manufacturer’s recommended protocol for binding and washing. The dried pellet was resuspended in Qiagen EB Buffer (55 ul), incubated at room temperature for 2 min, and then placed on the magnetic stand for 5 min. Supernatant (50 μl) was transferred to microcentrifuge tube for storage. The final amplicon pool was evaluated using the Agilent Fragment Analyzer automated electrophoresis system, quantified with the Qubit flourometer using the quant-iT HS dsDNA reagent kit (Invitrogen). 16S rRNA sequencing and ITS sequencing (2 × 250 paired-end sequencing) was performed on the Illumina MiSeq platform (Illumina Inc., San Diego, CA, USA).

### Bioinformatic processing and taxonomic assignment

All raw sequencing reads were processed using the DADA2 pipeline *ver* 1.16 (Callahan et al. 2016). Briefly, the amplification primer sequences were removed from all the reads, then truncated at 200 (forward) and 180 (reverse) base pairs, filtered by removing reads with any nonspecific sequences (NSs), residual phiX sequences, or an expected error higher than three. Quality filtered reads were denoised and merged, chimeric reads were removed, and ASVs were inferred. The resulting ASVs were assigned taxonomy (up to the species level) using the SILVA 138.1 database [55]. To identify fungal ASVs, the Cutadapt algorithm [56] was used to remove the primer sequences at both ends of the contigs and cull contigs that did not contain both primer sequences. DADA2 was used to perform similar quality filtering and ASV identification, and taxonomy was assigned as described above. Feature tables of ASVs were exported, and downstream microbiome analyses were performed using the Phyloseq package in R (McMurdie & Holmes, 2013).

Differences in mean raw read counts at different age points were analyzed using linear mixed models (LMM) with the *lmer* function from the lme4 R package (Bates et al., 2015). Fixed effect factors included housing types and cohort (cohort 1 vs 2), while pig ID was considered as a random effect. The effects of age, housing type, and cohort were assessed using statistical models with and without these factors, employing Restricted Maximum Likelihood. To determine the significance between age points in the final model, pairwise comparison tests were conducted using post hoc Tukey multiple comparison tests from the emmeans R package, with a significance level of *P* < 0.05. Additionally, to account for sequencing depth discrepancies, samples were normalized using CSS with default values [27].

### Phenotypic AMR of fecal bacteria

A total of 162 samples (*n* = 18 pigs per age point) were subjected to bacteriological culture to quantify coliforms and enterococci via spiral plating using an Eddy Jet 2 spiral plater (Neutech Group Inc., Farmingdale, NY, USA) as described previously [57]. Briefly, each fecal sample was diluted in phosphate-buffered saline (PBS) at a 1:10 ratio. The dilution was plated on MacConkey agar (MAC; Remel, Lenexa, KS, USA) and also on m-*Enterococcus* agar (ENT; Remel) to enumerate total coliform and enterococci counts respectively and on MAC and ENT plates supplemented with antimicrobial to enumerate those resistant to specific antimicrobials. Phenotypically AMR fecal coliforms or enterococci were determined by enumerating coliforms or enterococci counts in presence of the clinical break‐point concentration of an antimicrobial of that class.

To enumerate AMR coliform, eleven antimicrobials were selected for evaluation, representing nine different classes of antimicrobials: aminopenicillins (ampicillin, 16 µg/mL), aminoglycosides (gentamicin, 16 µg/mL; streptomycin, 32 µg/mL), 3rd-generation cephalosporins (ceftriaxone, 4 µg/mL), fluoroquinolones (ciprofloxacin, 1 µg/mL; enrofloxacin, 0.125 µg/mL), macrolides (azithromycin, 32 µg/mL), phenicols (chloramphenicol, 32 µg/mL), quinolones (nalidixic acid, 32 µg/mL), sulfonamide/folate pathway inhibitors (sulfamethoxazole, 512 µg/mL), and tetracyclines (tetracycline, 16 µg/mL).

Similarly to enumerate AMR enterococci, a total of elevan antimicrobials were selected for evaluation, representing eight different classes of antimicrobials: aminoglycosides (gentamicin, 500 µg/mL; streptomycin, 1024 µg/mL), fluoroquinolones (ciprofloxacin, 4 µg/mL; enrofloxacin, 4 µg/mL), lincosamides (lincomycin, 8 µg/mL), macrolides (erythromycin, 8 µg/mL; tylosin, 32 µg/mL), nitrofurans (nitrofurantoin, 128 µg/mL), penicillins (penicillin, 16 µg/mL), quinolones (nalidixic acid, 32 µg/mL) and tetracyclines (tetracycline, 16 µg/mL). The plates were incubated at 37 °C for 18 h for MAC and up to 48 h for ENT. The bacterial counts (CFU/g) were determined by counting the bacterial colonies and converting to log10 CFU/g feces for statistical analysis. Growth on plain agar plates (MAC or ENT) was compared to that on each antimicrobial-supplemented plate.

### Quantification of targeted AMR genes from metagenomic community DNA

The number of gene copies of *tet*(A), *bla*_CTX-M_, and 16S rRNA in the fecal community DNA per gram of wet feces (*n* = 8/age-point; 72 total samples, i.e., the same 72 samples that were used for fecal microbiome analysis) was estimated using quantitative real-time PCR (qPCR). The PCR plates were set up using a dedicated robot (QIAgility™, Qiagen, Valencia, CA), and DNA was used directly as a template in the qPCR reactions for quantification of the genes using an AriaMx real-time qPCR system (Agilent Technologies, La Jolla, CA). All qPCR assays were run in duplicate with two negative controls: one with a no-template control and the other with DNA-free water as a template. The primer set(s) and detailed methods for standard curve generation and quantification are described previously (Gaire et al. 2023). After each PCR run, data were extracted using AriaMx ver. 1.0 software (Agilent, Santa Clara, CA). The gene copy numbers per gram of wet feces were determined by adjusting the dilution factor for each step of DNA extraction. Gene copies were standardized by dividing the gene copy numbers by the 16S rRNA gene copies.

### Statistical analyses

Statistical analysis was performed to evaluate the temporal dynamics of fecal microbiome composition and AMR in the female pig cohorts. All analyses were performed in R statistical software (version 4.10). Figures were created using the ggplot2 package in R. In all the models, we considered *P* values < 0.05 to indicate statistical significance of the association. The fixed effects considered in all models were age (weeks) and cohort (1 *vs*. 2). The pig ID was specified as the random effect in all models to account for repeated measurement. For microbiome analysis, enterotypes, microbiome maturation score, alpha diversity matrices, beta diversity and relative abundance of individual bacterial genera were considered outcome variables. Similarly, the count of total and antimicrobial-resistant coliform or enterococci (log_10_-transformed CFU/g feces), quantities of the *bla*_CTX-M_ and *tet*(A) genes, as well as standardized *tet*(A) or *bla*_CTX-M_ gene copies, were considered as outcome variables for AMR analysis.

### Temporal dynamics of enterotypes and microbiome maturation score

Fecal bacterial enterotypes (i.e., microbial community types) were determined by fitting the bacterial genus counts using a Dirichlet multinomial mixture (DMM) model (Holmes et al. 2012). The number of enterotypes (i.e., community types) was chosen by selecting the number of DMM components that gave the lowest Laplace approximation to the negative log model. Each sample was assigned to an enterotype based on its maximum posterior probability. The proportion of samples occupying an enterotype at each age was determined, and changes in enterotypes over time were visualized [58].

To further evaluate whether fecal microbiome maturation coincided with enterotype, we estimated the microbiome maturation scores of the pigs as previously described by [28] based on the first appearance of genera (genera with relative abundance > 0.5%, representing 91% of total abundance) at different pig ages. We then ranked the genera based on the Kendall’s W test using the synchrony R package with 10,000 permutations, and median values of the rank were used to calculate the final order of the genera in each pig. A maturation score was calculated for each sample by averaging the ranks of the genera weighted by the presence or absence of a specific genus.

### Alpha diversity

The alpha diversity of the microbiome, measured as the richness (number of unique features per sample) and Shannon diversity (accounting for both richness and evenness in the abundance of features), was estimated for each sample at ASV level using the *alpha* function in the microbiome package in R. To evaluate the changes in mean values for each alpha diversity metric (outcome) across different age points (fixed effect), we fitted linear mixed models separately for each alpha diversity metric using the linear mixed effect models using *lmer* function (lme4 R package). We also included the number of raw reads in the model as a fixed effect due to the differences observed in the average number of raw reads across age points. To test for statistical significance between age points in the final model, post hoc pairwise comparisons of significant fixed effects were determined using the *emmeans* package (version 1.3.4), resulting in *P* values that were Tukey adjusted for multiplicity as part of the emmeans workflow.

### Beta diversity

The differences in overall fecal bacterial community composition (beta diversity) between samples were assessed based on the Bray‒Curtis distance matrix calculated from the normalized ASV count matrices, as well at other taxonomic levels including at phylum, class, family and genus. To visualize the overall bacterial composition by age and physiological stage (i.e., early age, growing, estrus, parturition and weaning), nonmetric multidimensional scaling (NMDS) ordination plots were generated based on the calculated Bray‒Curtis dissimilarity value using the *metamds* function in R. To test differences in overall bacterial composition by age point, we used permutational multivariate analysis of variance (PERMANOVA) using the adonis2 function (vegan package in R) with 10,000 permutations. We treated the distance metrics as the outcome variable and the age and cohort (1 *vs*. 2) while controlling the number of raw sequences (as continuous terms). We accounted for the clustering of data due to the repeated sampling of the pigs by adding the *strata* option within the adonis2 function. The R^2^ value and *P* values obtained from the model(s) were used to describe the percentage of the microbial community structure variation that was explained by each explanatory variable and the corresponding statistical significance. If a significant result (*P* < 0.05) was observed, we performed pairwise comparisons using the *pairwise.adonis* function.

To assess the divergence in bacterial composition from 3 weeks of age to gestation and weaning of the first litter, we fitted the linear mixed models with Bray‒Curtis dissimilarity values (outcome variable) and age and cohort (1 *vs*. 2) as fixed effects and pig identity as a random effect to account for repeated measures with a similar model structure to that described above. Similarly, differences in overall fecal fungal diversity and community composition by age were analyzed using linear mixed effect models and PERMANOVA as described above.

### Temporal dynamics of relative abundance of bacterial genera

To determine the longitudinal changes in individual bacterial genera abundance associated with age, differential abundance testing was performed using the *Maaslin2* function in R [59]. The CSS-normalized microbial count matrices were the input data, and the default Maaslin2 parameters were applied (minimum percentage relative abundance 0.01%, *P* < 0.05, q < 0.25, LMM models). All *P* values were adjusted for multiple comparisons using the Benjamini–Hochberg false discovery rate (FDR) [60]. We then grouped bacterial genera based on their change in abundance having (i) significantly increased, (ii) significantly decreased, or (iii) no significant change from week 3 of life to week 53 of life.

### Phenotypic AMR and quantification of targeted AMR genes

The changes in the AMR coliform and enterococcus count (expressed as Log_10_-transformed CFU/g feces) data exhibited substantial variation over time. Thus, we fitted semiparametric generalized additive regression models (generalized additive models, GAMs) (the “mgcv” package in R) that allowed smooth trends to be estimated from the data using regression splines. We included age as a continuous smooth term and cohort (1 or 2) as a linear parametric term. We added a continuous-time AR(1) correlation structure to account for temporal autocorrelation in the residuals between time points. We then computed the standard error for the first derivatives of the parameter and the 95% pointwise confidence interval (CI) on the derivative. The periods of significance changing age were determined when the 95% CI on the first derivatives was bounded away from zero. Because neither coliforms nor enterococci grew in the presence of fluoroquinolones (ciprofloxacin, 4 µg/mL; enrofloxacin, 4 µg/mL) and coliforms alone did not grow in the presence of quinolones (nalidixic acid, 32 µg/mL), these bacteria were excluded from the dataset and subsequent analyses.

Similarly, for targeted AMR genes, each of the *bla*_CTX-M_ and *tet*(A) gene copies as well as standardized *tet*(A) or *bla*_CTX-M_ gene copies (i.e., normalized to 16S rRNA gene copies) in each gram of feces were logarithmically transformed to base 10 for use as an outcome variable in linear mixed models. The linear mixed models were used to evaluate the effects of age on two outcomes: (a) absolute (non-standardized) gene copy numbers per gram of feces and (b) standardized quantities of the genes normalized to 16S rRNA gene copies in the original fecal samples.

### Supplementary Information

**Additional file 1**. **Table S1**: Metadata and 16S rRNA sequencing reads for all samples included in this study.

**Additional File 2**. **Figure S1**: A-D: A) Model fit for the number of Dirichlet mixture components (K) (DMM clusters) using the Laplace approximation to the negative log model. B) NMDS plot showing overall microbial composition with enterotypes (DMM 1-4 represent Enterotypes), C-D) Alpha diversity values (richness and Shannon’s diversity, respectively) per each DMM cluster/Enterotype.

**Additional File 3**. **Figure S2**: Non-metric multidimensional scaling (NMDS) ordinating plot based on Bray-Curtis distances illustrates variation in microbial community structures by stages at: A-D) Phylum, Class, Family and Genus levels and E-H) Beta-dispersion values (distance to centroid) for each age group and for each respective taxonomic level. R^2^ represents the amount of variability explained by the stages and the associated P-value is based on PERMANOVA analysis.

**Additional File 4**. **Figure S3**: Relative abundance of the top 15 bacterial genera across pig age.

**Additional File 5**. **Table S2**: Complete list of microbial features (genera) whose abundances were associated with age of pig (in weeks) as determined by the Maaslin 2 (Microbiome Multivariable Associations with Linear Models) approach.

**Additional File 6**. **Figure S4**: Relative abundance of fungi at A) Phylum and B) Family levels, and C) distance to centroid (beta dispersion of overall fecal fungi composition at genus level) for each age point.

**Additional File 7**. **Figure S5**: Mean changes in (A) nonstandardized log_10_ *tet*(A), (B) nonstandardized log_10_ *bla*_CTX-M_, (C) standardized (to 16S rRNA) log_10_ *tet*(A), and (D) standardized (to 16S rRNA) log_10_ *bla*_CTX-M_ log_10_ gene copy numbers per gram wet feces over time (pig age in weeks), and (E) Total log_10_ 16S rRNA gene copies per gram wet feces over time (pig age in weeks).

## Data Availability

The raw sequence data generated from amplicon sequencing during this study is available in the NCBI repository under BioProject ID PRJNA97705. Metadata for all samples included in this study are presented in Additional file 1. The source data and codes related to the manuscript codes have been deposited in the GitHub repository (https://github.com/tngaire/microbiome_amr_female_pigs).
