# Supplementary material for: Temporal dynamics of the fecal microbiome in female pigs from early life through estrus, parturition, and weaning of the first litter of piglets
Source: Anim Microbiome. 2024 Feb 21;6:7. doi: 10.1186/s42523-024-00294-8 (PMC10882843; doi:10.1186/s42523-024-00294-8)
Supplement: Supplementary file 1 — Additional file 1. Table S1: Metadata and 16S rRNA sequencing reads for all samples included in this study. [file 42523_2024_294_MOESM1_ESM.docx]

## Additional Files

## Temporal dynamics of the fecal microbiome in female pigs from early life through estrus, parturition, and weaning of the first litter of piglets

## Tara N. Gaire^1^, H. Morgan Scott^2^, Noelle R. Noyes^3^, Aaron C. Ericsson^4^, Michael D. Tokach^5^, Hayden William^5^, Mariana B. Menegat^5^, Javier Vinasco^2^, T. G. Nagaraja^1^, Victoriya V. Volkova^1^

^1^Department of Diagnostic Medicine/Pathobiology, College of Veterinary Medicine, Kansas State University, Manhattan, KS, 66506, USA

^2^Department of Veterinary Pathobiology, School of Veterinary Medicine and Biomedical Sciences, Texas A&M University, College Station, TX, 77843, USA

^3^Department of Veterinary Population Medicine, College of Veterinary Medicine, University of Minnesota, St. Paul, MN, 55108, USA

^4^Department of Veterinary Pathobiology, College of Veterinary Medicine, University of Missouri, MO, 65211, USA

^5^Department of Animal Sciences and Industry, College of Agriculture, Kansas State University, Manhattan, KS, 66506, USA

Corresponding author:

T. G. Nagaraja ([tnagaraj@vet.k-state.edu](mailto:tnagaraj@vet.k-state.edu))

**Additional file 1: Table S1**. Metadata and 16S rRNA sequencing reads for all samples included in this study

| SampleID | S.Name | PigID | Age, weeks | Input reads | Filtered | Denoised  Forward | Denoised Reverse | Merged | Nonchimeric |
| --- | --- | --- | --- | --- | --- | --- | --- | --- | --- |
| VV073.A1 | VV073 | 292 | 3 | 34519 | 31843 | 31480 | 31025 | 29714 | 29573 |
| VV074.A2 | VV074 | 296 | 3 | 130784 | 121382 | 119955 | 119143 | 108748 | 97178 |
| VV075.A3 | VV075 | 389 | 3 | 148671 | 138336 | 135568 | 133903 | 118404 | 105234 |
| VV076.A4 | VV076 | 391 | 3 | 162638 | 149808 | 146614 | 143694 | 126437 | 111605 |
| VV077.A5 | VV077 | 363 | 3 | 143400 | 132857 | 130914 | 129590 | 118417 | 103335 |
| VV078.A6 | VV078 | 366 | 3 | 824 | 747 | 692 | 675 | 586 | 586 |
| VV079.A7 | VV079 | 369 | 3 | 142823 | 132462 | 130380 | 129393 | 114296 | 93785 |
| VV080.A8 | VV080 | 379 | 3 | 85086 | 78117 | 76864 | 76184 | 74326 | 73800 |
| VV081.A9 | VV081 | 292 | 6 | 177169 | 163390 | 160335 | 159439 | 141562 | 122710 |
| VV082.A10 | VV082 | 296 | 6 | 144475 | 133163 | 131158 | 129822 | 116146 | 101787 |
| VV083.A11 | VV083 | 389 | 6 | 163031 | 151061 | 148166 | 146660 | 127285 | 109346 |
| VV084.A12 | VV084 | 391 | 6 | 148294 | 136660 | 134436 | 132882 | 117515 | 103864 |
| VV085.B1 | VV085 | 363 | 6 | 118474 | 110251 | 107701 | 106278 | 91017 | 80022 |
| VV086.B2 | VV086 | 366 | 6 | 184222 | 168940 | 165705 | 164434 | 145388 | 122224 |
| VV087.B3 | VV087 | 369 | 6 | 166438 | 153127 | 150644 | 149113 | 135061 | 119029 |
| VV088.B4 | VV088 | 379 | 6 | 161940 | 150144 | 147124 | 145297 | 125630 | 108829 |
| VV089.B5 | VV089 | 292 | 10 | 159394 | 146935 | 144025 | 142099 | 125048 | 110846 |
| VV090.B6 | VV090 | 296 | 10 | 172962 | 158812 | 155420 | 153703 | 134532 | 116415 |
| VV091.B7 | VV091 | 389 | 10 | 159957 | 147484 | 144679 | 142671 | 123467 | 100592 |
| VV092.B8 | VV092 | 391 | 10 | 173189 | 159450 | 156484 | 154421 | 134954 | 114314 |
| VV093.B9 | VV093 | 363 | 10 | 169092 | 155419 | 151730 | 149826 | 127480 | 106820 |
| VV094.B10 | VV094 | 366 | 10 | 156873 | 145344 | 142699 | 140553 | 121671 | 102092 |
| VV095.B11 | VV095 | 369 | 10 | 156481 | 144048 | 140845 | 139258 | 120193 | 101094 |
| VV096.B12 | VV096 | 379 | 10 | 152090 | 140438 | 137908 | 136319 | 118956 | 100683 |
| VV097.C1 | VV097 | 292 | 12 | 176392 | 162072 | 158732 | 157255 | 137451 | 113636 |
| VV098.C2 | VV098 | 296 | 12 | 183844 | 168589 | 165610 | 164497 | 144794 | 118811 |
| VV099.C3 | VV099 | 389 | 12 | 160077 | 146641 | 143651 | 142034 | 124522 | 107313 |
| VV100.C4 | VV100 | 391 | 12 | 161010 | 148167 | 144768 | 143257 | 124468 | 105955 |
| VV101.C5 | VV101 | 363 | 12 | 184089 | 169050 | 165757 | 164302 | 143024 | 115655 |
| VV102.C6 | VV102 | 366 | 12 | 162843 | 149833 | 146531 | 144963 | 126377 | 108224 |
| VV103.C7 | VV103 | 369 | 12 | 177228 | 163647 | 160322 | 158682 | 138501 | 115165 |
| VV104.C8 | VV104 | 379 | 12 | 172025 | 157458 | 154360 | 152464 | 133020 | 111684 |
| VV105.C9 | VV105 | 292 | 22 | 160153 | 146394 | 142788 | 141203 | 120008 | 100473 |
| VV106.C10 | VV106 | 296 | 22 | 153110 | 141767 | 138323 | 135844 | 115266 | 101487 |
| VV107.C11 | VV107 | 389 | 22 | 151643 | 140313 | 137358 | 135419 | 117194 | 99433 |
| VV108.C12 | VV108 | 391 | 22 | 118810 | 109335 | 106813 | 104986 | 91486 | 82930 |
| VV109.D1 | VV109 | 363 | 22 | 156765 | 145015 | 141995 | 140268 | 121404 | 104429 |
| VV110.D2 | VV110 | 366 | 22 | 149415 | 138143 | 134726 | 133305 | 114791 | 101782 |
| VV111.D3 | VV111 | 369 | 22 | 156100 | 144845 | 141191 | 139157 | 118849 | 103156 |
| VV112.D4 | VV112 | 379 | 22 | 167218 | 153913 | 150959 | 149615 | 131753 | 110322 |
| VV113.D5 | VV113 | 292 | 32 | 145914 | 135589 | 132639 | 130493 | 112082 | 94330 |
| VV114.D6 | VV114 | 296 | 32 | 167535 | 153977 | 149912 | 147414 | 125305 | 106546 |
| VV115.D7 | VV115 | 389 | 32 | 158592 | 147615 | 144000 | 142421 | 121446 | 98101 |
| VV116.D8 | VV116 | 391 | 32 | 150030 | 139220 | 135992 | 133719 | 114372 | 98672 |
| VV117.D9 | VV117 | 363 | 32 | 166815 | 154067 | 150179 | 148360 | 125231 | 105734 |
| VV118.D10 | VV118 | 366 | 32 | 141885 | 131891 | 128694 | 126917 | 107169 | 92707 |
| VV119.D11 | VV119 | 369 | 32 | 139365 | 130285 | 127324 | 125785 | 108392 | 89317 |
| VV120.D12 | VV120 | 379 | 32 | 150261 | 139409 | 136080 | 133930 | 114504 | 97082 |
| VV121.E1 | VV121 | 292 | 49 | 168610 | 155118 | 151047 | 149310 | 125602 | 101501 |
| VV122.E2 | VV122 | 296 | 49 | 145031 | 133603 | 130489 | 129551 | 112353 | 93903 |
| VV123.E3 | VV123 | 389 | 49 | 164945 | 153662 | 150329 | 147595 | 127199 | 112124 |
| VV124.E4 | VV124 | 391 | 49 | 158383 | 147100 | 143602 | 141671 | 121721 | 105325 |
| VV125.E5 | VV125 | 363 | 49 | 170028 | 157368 | 153675 | 151151 | 128955 | 105780 |
| VV126.E6 | VV126 | 366 | 49 | 177098 | 162012 | 158745 | 157331 | 138504 | 117794 |
| VV127.E7 | VV127 | 369 | 49 | 180053 | 165384 | 161679 | 159711 | 137941 | 118098 |
| VV128.E8 | VV128 | 379 | 49 | 145794 | 135503 | 132724 | 131310 | 115889 | 101105 |
| VV129.E9 | VV129 | 292 | 50 | 160527 | 148185 | 145143 | 143202 | 124308 | 106087 |
| VV130.E10 | VV130 | 296 | 50 | 142549 | 131813 | 129388 | 127957 | 114525 | 100471 |
| VV131.E11 | VV131 | 389 | 50 | 140201 | 129858 | 126975 | 124792 | 107163 | 93480 |
| VV132.E12 | VV132 | 391 | 50 | 158559 | 146709 | 143328 | 141159 | 121119 | 106897 |
| VV133.F1 | VV133 | 363 | 50 | 143601 | 130898 | 127424 | 125356 | 107003 | 94713 |
| VV134.F2 | VV134 | 366 | 50 | 141990 | 130982 | 127324 | 125230 | 104468 | 89524 |
| VV135.F3 | VV135 | 369 | 50 | 143913 | 132510 | 129152 | 126940 | 108578 | 95292 |
| VV136.F4 | VV136 | 379 | 50 | 146094 | 134928 | 131454 | 128820 | 109327 | 94388 |
| VV137.F5 | VV137 | 292 | 53 | 147395 | 135043 | 132097 | 130474 | 115191 | 97420 |
| VV138.F6 | VV138 | 296 | 53 | 169281 | 155246 | 151954 | 150096 | 131886 | 109368 |
| VV139.F7 | VV139 | 389 | 53 | 165092 | 150426 | 147264 | 145372 | 127188 | 108038 |
| VV140.F8 | VV140 | 391 | 53 | 149635 | 137323 | 134789 | 133148 | 119033 | 104236 |
| VV141.F9 | VV141 | 363 | 53 | 162322 | 148311 | 144649 | 142091 | 121991 | 108081 |
| VV142.F10 | VV142 | 366 | 53 | 134822 | 124314 | 121318 | 119000 | 101303 | 90306 |
| VV143.F11 | VV143 | 369 | 53 | 130335 | 120035 | 117466 | 115644 | 101567 | 91707 |
| VV144.F12 | VV144 | 379 | 53 | 151880 | 139766 | 136376 | 133509 | 113621 | 99241 |
