# Supplementary material for: Temporal dynamics of the fecal microbiome in female pigs from early life through estrus, parturition, and weaning of the first litter of piglets
Source: Anim Microbiome. 2024 Feb 21;6:7. doi: 10.1186/s42523-024-00294-8 (PMC10882843; doi:10.1186/s42523-024-00294-8)
Supplement: Supplementary file 2 — Additional File 2. Figure S1: A-D: A) Model fit for the number of Dirichlet mixture components (K) (DMM clusters) using the Laplace approximation to the negative log model. B) NMDS plot showing overall microbial composition with enterotypes (DMM 1-4 represent Enterotypes), C-D) Alpha diversity values (richness and Shannon’s diversity, respectively) per each DMM cluster/Enterotype. [file 42523_2024_294_MOESM2_ESM.docx]

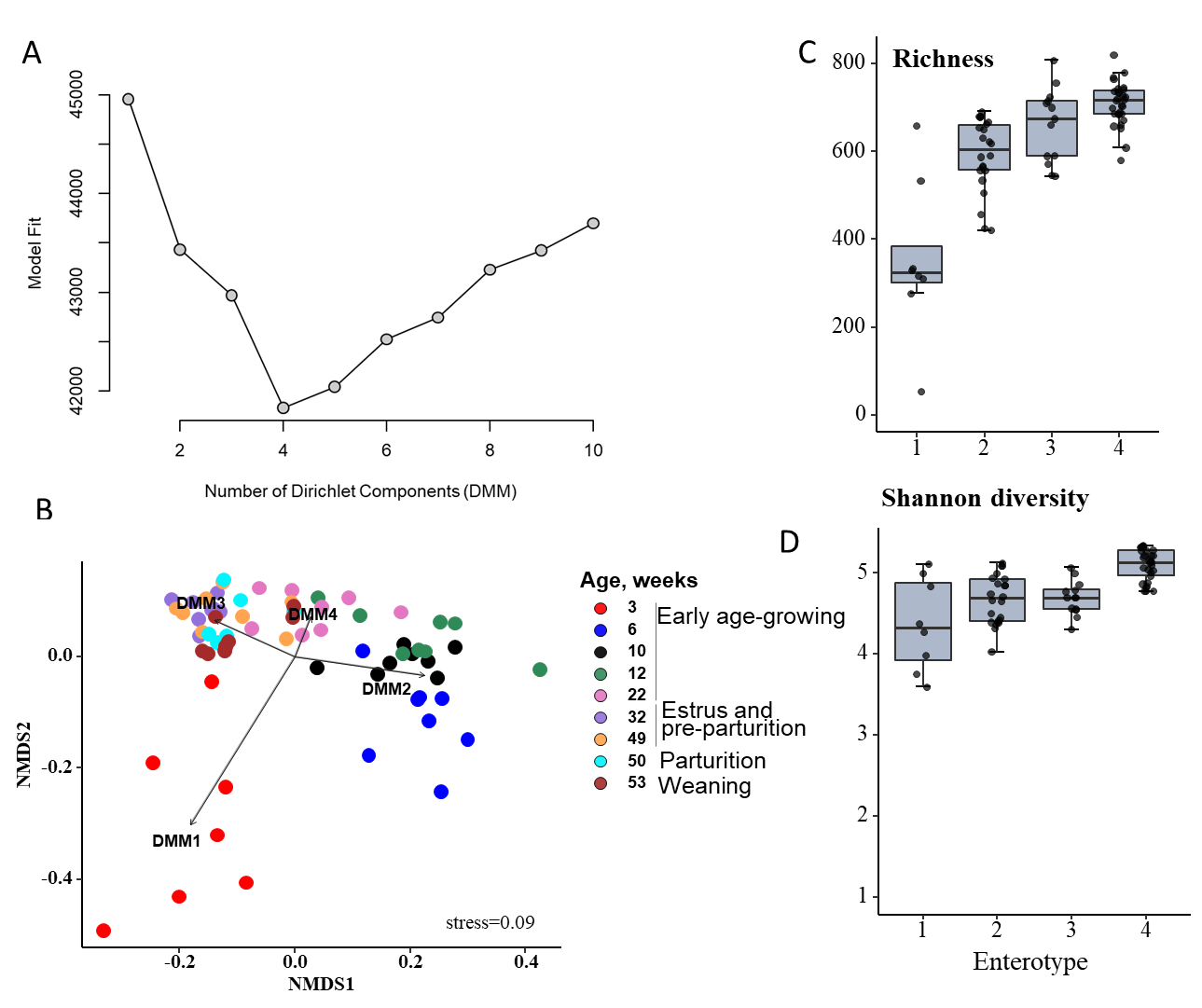


**Additional File 2. FigS1 A-D. A)** Model fit for the number of Dirichlet mixture components (K) (DMM clusters) using the Laplace approximation to the negative log model. B) NMDS plot showing overall microbial composition with enterotypes (DMM 1-4 represent Enterotypes), C-D) Alpha diversity values (richness and Shannon’s diversity, respectively) per each DMM cluster/Enterotype.
