# Supplementary material for: Temporal dynamics of the fecal microbiome in female pigs from early life through estrus, parturition, and weaning of the first litter of piglets
Source: Anim Microbiome. 2024 Feb 21;6:7. doi: 10.1186/s42523-024-00294-8 (PMC10882843; doi:10.1186/s42523-024-00294-8)
Supplement: Supplementary file 3 — Additional File 3. Figure S2: Non-metric multidimensional scaling (NMDS) ordinating plot based on Bray-Curtis distances illustrates variation in microbial community structures by stages at: A-D) Phylum, Class, Family and Genus levels and E-H) Beta-dispersion values (distance to centroid) for each age group and for each respective taxonomic level. R2 represents the amount of variability explained by the stages and the associated P-value is based on PERMANOVA analysis. [file 42523_2024_294_MOESM3_ESM.docx]

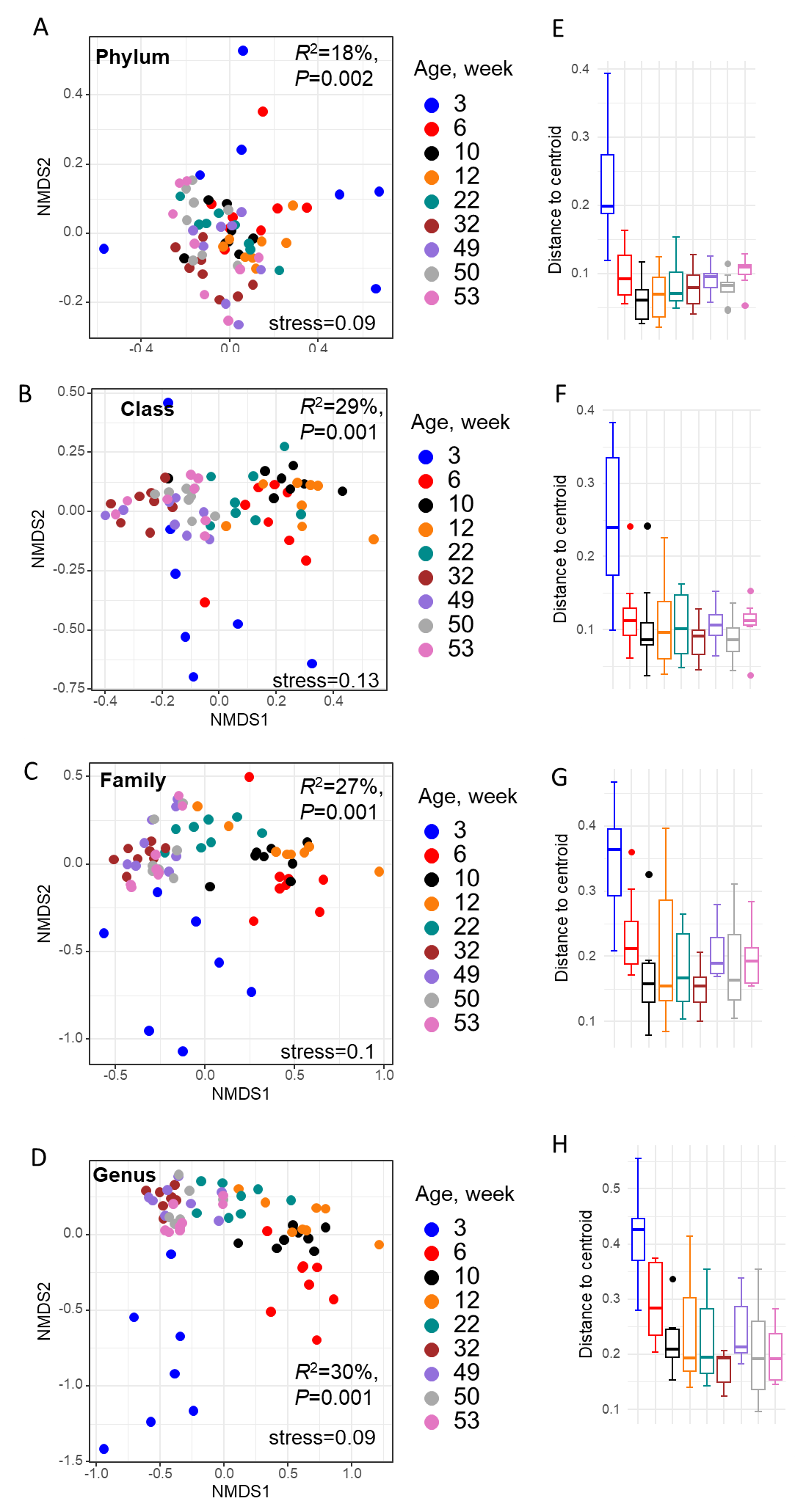


**Additional File 3. FigS2.** Non-metric multidimensional scaling (NMDS) ordinating plot based on Bray-Curtis distances illustrates variation in microbial community structures by stages at: A-D) Phylum, Class, Family and Genus levels and E-H) Beta-dispersion values (distance to centroid) for each age group and for each respective taxonomic level. *R^2^* represents the amount of variability explained by the stages and the associated *P*-value is based on PERMANOVA analysis.
