# Supplementary figures and images for: Temporal dynamics of the fecal microbiome in female pigs from early life through estrus, parturition, and weaning of the first litter of piglets
Source: Anim Microbiome. 2024 Feb 21;6:7. doi: 10.1186/s42523-024-00294-8 (PMC10882843; doi:10.1186/s42523-024-00294-8)

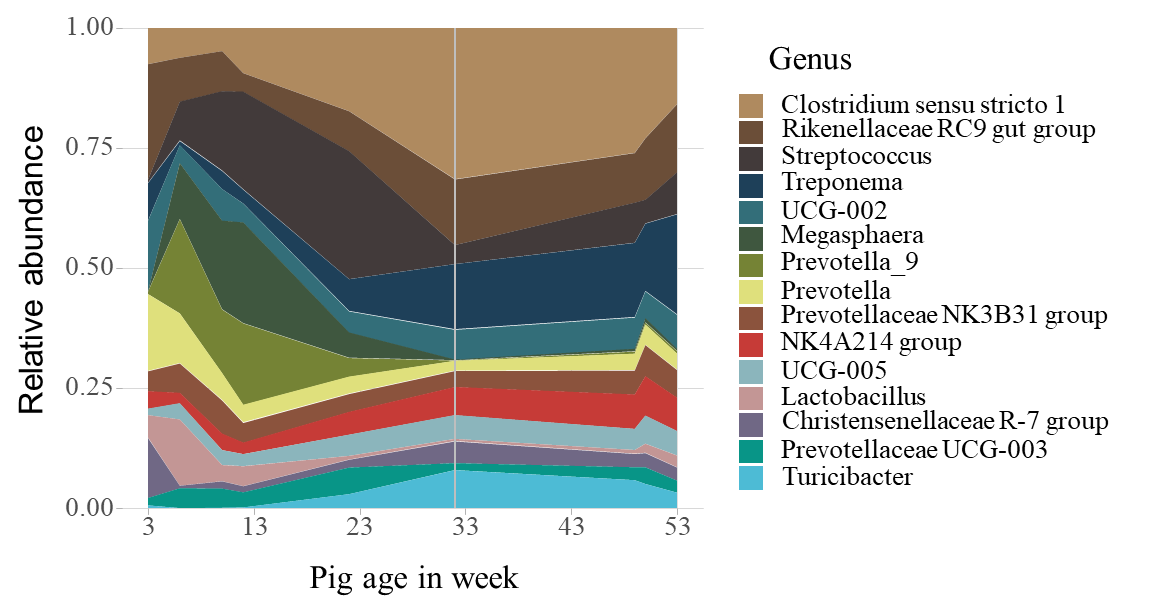


**Additional File 4. FigS3.** Relative abundance of the top 15 bacterial genera across pig age.

Supplement: Supplementary file 4 — Additional File 4. Figure S3: Relative abundance of the top 15 bacterial genera across pig age. [file 42523_2024_294_MOESM4_ESM.docx]
