# Supplementary material for: Temporal dynamics of the fecal microbiome in female pigs from early life through estrus, parturition, and weaning of the first litter of piglets
Source: Anim Microbiome. 2024 Feb 21;6:7. doi: 10.1186/s42523-024-00294-8 (PMC10882843; doi:10.1186/s42523-024-00294-8)
Supplement: Supplementary file 5 — Additional File 5. Table S2: Complete list of microbial features (genera) whose abundances were associated with age of pig (in weeks) as determined by the Maaslin 2 (Microbiome Multivariable Associations with Linear Models) approach. [file 42523_2024_294_MOESM5_ESM.docx]

**Additional File 5: Table S2**. Complete list of microbial features (genera) whose abundances were associated with age of pig (in weeks) as determined by the MaAsLin 2 (Microbiome Multivariable Associations with Linear Models) approach.

| Feature (genus) | coef | stderr | N | N.not.0 | pval | qval | trajectories |
| --- | --- | --- | --- | --- | --- | --- | --- |
| Prevotella_7 | -0.13274 | 0.019947 | 72 | 45 | 5.29E-09 | 3.20E-08 | decrease |
| Intestinimonas | -0.10801 | 0.008799 | 72 | 56 | 4.02E-19 | 4.87E-17 | decrease |
| Ligilactobacillus | -0.10721 | 0.016107 | 72 | 49 | 5.26E-09 | 3.20E-08 | decrease |
| Agathobacter | -0.09832 | 0.015016 | 72 | 35 | 8.24E-09 | 4.75E-08 | decrease |
| Blautia | -0.09729 | 0.014316 | 72 | 57 | 4.52E-09 | 3.04E-08 | decrease |
| HT002 | -0.09723 | 0.012309 | 72 | 43 | 2.80E-11 | 2.60E-10 | decrease |
| Chlamydia | -0.09415 | 0.013463 | 72 | 30 | 1.29E-09 | 9.72E-09 | decrease |
| Dorea | -0.08894 | 0.010154 | 72 | 46 | 7.30E-13 | 1.10E-11 | decrease |
| Holdemanella | -0.08592 | 0.014519 | 72 | 48 | 1.09E-07 | 4.56E-07 | decrease |
| UCG.003 | -0.07872 | 0.011018 | 72 | 39 | 1.12E-09 | 9.03E-09 | decrease |
| Prevotella_9 | -0.07449 | 0.01632 | 72 | 67 | 2.09E-05 | 5.49E-05 | decrease |
| Dialister | -0.07414 | 0.020918 | 72 | 50 | 0.000706 | 0.001294 | decrease |
| Clostridium.sensu.stricto.6 | -0.07381 | 0.015534 | 72 | 51 | 1.04E-05 | 2.92E-05 | decrease |
| Faecalibacterium | -0.07298 | 0.016169 | 72 | 56 | 2.51E-05 | 6.32E-05 | decrease |
| Collinsella | -0.07272 | 0.012852 | 72 | 51 | 3.11E-07 | 1.07E-06 | decrease |
| Lachnospiraceae.NK4A136.group | -0.07016 | 0.008593 | 72 | 69 | 1.84E-11 | 1.86E-10 | decrease |
| X.Eubacterium..eligens.group | -0.07013 | 0.013445 | 72 | 34 | 1.77E-06 | 5.64E-06 | decrease |
| Fournierella | -0.06855 | 0.014774 | 72 | 51 | 1.58E-05 | 4.25E-05 | decrease |
| Shuttleworthia | -0.06429 | 0.014795 | 72 | 43 | 4.61E-05 | 0.000112 | decrease |
| Subdoligranulum | -0.06323 | 0.018865 | 72 | 63 | 0.001297 | 0.002343 | decrease |
| X.Eubacterium..xylanophilum.group | -0.06175 | 0.013009 | 72 | 40 | 1.06E-05 | 2.92E-05 | decrease |
| CAG.873 | -0.05921 | 0.011482 | 72 | 19 | 2.23E-06 | 6.93E-06 | decrease |
| Erysipelotrichaceae.UCG.006 | -0.05809 | 0.00907 | 72 | 29 | 1.50E-08 | 7.24E-08 | decrease |
| Cloacibacillus | -0.05672 | 0.017036 | 72 | 17 | 0.001391 | 0.002439 | decrease |
| Lachnospiraceae.FCS020.group | -0.05338 | 0.010368 | 72 | 49 | 2.79E-06 | 8.43E-06 | decrease |
| Veillonella | -0.05265 | 0.011929 | 72 | 16 | 3.60E-05 | 8.90E-05 | decrease |
| Catenibacterium | -0.05097 | 0.017161 | 72 | 32 | 0.004079 | 0.006761 | decrease |
| Anaerostipes | -0.05001 | 0.009401 | 72 | 22 | 1.18E-06 | 3.87E-06 | decrease |
| Lactobacillus | -0.04952 | 0.014978 | 72 | 72 | 0.001564 | 0.002704 | decrease |
| Sutterella | -0.04738 | 0.012879 | 72 | 60 | 0.000457 | 0.000937 | decrease |
| X.Ruminococcus..torques.group | -0.04577 | 0.012654 | 72 | 28 | 0.000559 | 0.001108 | decrease |
| Intestinibacter | -0.04528 | 0.007762 | 72 | 64 | 1.54E-07 | 5.82E-07 | decrease |
| X.Ruminococcus..gauvreauii.group | -0.04451 | 0.011371 | 72 | 39 | 0.000208 | 0.000458 | decrease |
| Acidaminococcus | -0.0442 | 0.015616 | 72 | 49 | 0.006063 | 0.009286 | decrease |
| Megasphaera | -0.04018 | 0.023035 | 72 | 66 | 0.085523 | 0.10893 | decrease |
| Mogibacterium | -0.03821 | 0.009601 | 72 | 66 | 0.000181 | 0.000405 | decrease |
| Alistipes | -0.03819 | 0.017717 | 72 | 45 | 0.034567 | 0.04753 | decrease |
| Eisenbergiella | -0.03762 | 0.011276 | 72 | 22 | 0.001359 | 0.002419 | decrease |
| Colidextribacter | -0.03513 | 0.009845 | 72 | 71 | 0.000654 | 0.001236 | decrease |
| X.Eubacterium..ruminantium.group | -0.03495 | 0.015256 | 72 | 52 | 0.025325 | 0.035631 | decrease |
| Butyricicoccus | -0.03488 | 0.008432 | 72 | 61 | 9.65E-05 | 0.000229 | decrease |
| Alloprevotella | -0.03438 | 0.008413 | 72 | 72 | 0.000115 | 0.000267 | decrease |
| X.Eubacterium..hallii.group | -0.03393 | 0.011689 | 72 | 67 | 0.004947 | 0.008089 | decrease |
| Sharpea | -0.03351 | 0.015384 | 72 | 59 | 0.032777 | 0.045586 | decrease |
| Catenisphaera | -0.02808 | 0.013318 | 72 | 54 | 0.038595 | 0.051318 | decrease |
| Lachnoclostridium | -0.02714 | 0.013277 | 72 | 64 | 0.044719 | 0.057564 | decrease |
| Marvinbryantia | -0.02658 | 0.009244 | 72 | 57 | 0.005492 | 0.00863 | decrease |
| Streptococcus | -0.02446 | 0.016166 | 72 | 71 | 0.134744 | 0.168083 | decrease |
| Limosilactobacillus | -0.0241 | 0.01698 | 72 | 62 | 0.160806 | 0.194575 | decrease |
| Anaerovibrio | -0.02403 | 0.015142 | 72 | 66 | 0.117045 | 0.147525 | decrease |
| Selenomonas | -0.0212 | 0.017083 | 72 | 42 | 0.21912 | 0.257413 | decrease |
| Fusobacterium | -0.01863 | 0.014079 | 72 | 22 | 0.190094 | 0.227736 | decrease |
| Incertae.Sedis | -0.01846 | 0.007533 | 72 | 71 | 0.016749 | 0.024127 | decrease |
| Prevotella | -0.01767 | 0.005734 | 72 | 72 | 0.002939 | 0.005008 | decrease |
| Roseburia | -0.01607 | 0.011224 | 72 | 69 | 0.156744 | 0.191576 | decrease |
| Coprococcus | -0.01463 | 0.011311 | 72 | 67 | 0.200124 | 0.237402 | decrease |
| Solobacterium | -0.0095 | 0.014218 | 72 | 62 | 0.50602 | 0.541845 | decrease |
| Oribacterium | -0.00923 | 0.009527 | 72 | 70 | 0.335842 | 0.379784 | decrease |
| Phascolarctobacterium | -0.0047 | 0.006174 | 72 | 72 | 0.448743 | 0.493618 | decrease |
| Oxalobacter | -0.00199 | 0.013185 | 72 | 56 | 0.88046 | 0.918411 | decrease |
| Olsenella | -0.00079 | 0.015375 | 72 | 53 | 0.959095 | 0.965273 | decrease |
| Pyramidobacter | -0.00068 | 0.015465 | 72 | 55 | 0.965273 | 0.965273 | decrease |
| Lachnospira | -0.0005 | 0.010549 | 72 | 63 | 0.96222 | 0.965273 | decrease |
| X.Eubacterium..nodatum.group | 0.00111 | 0.013507 | 72 | 50 | 0.934732 | 0.958497 | increase |
| Succinivibrio | 0.00162 | 0.013131 | 72 | 70 | 0.902228 | 0.933074 | increase |
| Helicobacter | 0.004274 | 0.013555 | 72 | 54 | 0.753557 | 0.799829 | increase |
| Campylobacter | 0.004276 | 0.014378 | 72 | 68 | 0.767023 | 0.807041 | increase |
| Oscillospira | 0.005912 | 0.007948 | 72 | 72 | 0.459694 | 0.501108 | increase |
| Prevotellaceae.UCG.003 | 0.007866 | 0.009706 | 72 | 72 | 0.420431 | 0.466717 | increase |
| UCG.004 | 0.008156 | 0.007753 | 72 | 71 | 0.296404 | 0.338349 | increase |
| Peptococcus | 0.008657 | 0.010664 | 72 | 61 | 0.41995 | 0.466717 | increase |
| Escherichia.Shigella | 0.012333 | 0.017595 | 72 | 65 | 0.485666 | 0.524693 | increase |
| UCG.002 | 0.013579 | 0.006482 | 72 | 72 | 0.040213 | 0.052889 | increase |
| Rikenellaceae.RC9.gut.group | 0.013698 | 0.005205 | 72 | 72 | 0.010451 | 0.015612 | increase |
| Sphaerochaeta | 0.013893 | 0.00929 | 72 | 72 | 0.139262 | 0.171946 | increase |
| Prevotellaceae.NK3B31.group | 0.014711 | 0.005099 | 72 | 72 | 0.005194 | 0.008297 | increase |
| Corynebacterium | 0.016072 | 0.014884 | 72 | 27 | 0.283948 | 0.327216 | increase |
| Mitsuokella | 0.01837 | 0.01681 | 72 | 59 | 0.278233 | 0.323714 | increase |
| Monoglobus | 0.021536 | 0.007654 | 72 | 71 | 0.006352 | 0.009607 | increase |
| Parabacteroides | 0.021958 | 0.010256 | 72 | 70 | 0.035763 | 0.048621 | increase |
| Christensenellaceae.R.7.group | 0.022359 | 0.008725 | 72 | 72 | 0.012538 | 0.018501 | increase |
| Ruminococcus | 0.024127 | 0.011768 | 72 | 70 | 0.044093 | 0.057368 | increase |
| Desulfovibrio | 0.02832 | 0.009938 | 72 | 70 | 0.005743 | 0.008909 | increase |
| X.Eubacterium..siraeum.group | 0.03151 | 0.014855 | 72 | 62 | 0.037455 | 0.050356 | increase |
| Prevotellaceae.UCG.004 | 0.034158 | 0.011801 | 72 | 69 | 0.005211 | 0.008297 | increase |
| Anaerosporobacter | 0.034987 | 0.00968 | 72 | 38 | 0.000599 | 0.001169 | increase |
| Bifidobacterium | 0.035406 | 0.011921 | 72 | 64 | 0.004077 | 0.006761 | increase |
| Oscillibacter | 0.037009 | 0.009471 | 72 | 71 | 0.00023 | 0.000497 | increase |
| UCG.008 | 0.038238 | 0.015945 | 72 | 39 | 0.019152 | 0.027263 | increase |
| UCG.007 | 0.041711 | 0.007632 | 72 | 28 | 8.41E-07 | 2.83E-06 | increase |
| UCG.009 | 0.042212 | 0.007225 | 72 | 71 | 1.48E-07 | 5.82E-07 | increase |
| NK4A214.group | 0.042233 | 0.00449 | 72 | 71 | 1.29E-13 | 2.23E-12 | increase |
| Z20 | 0.042932 | 0.016865 | 72 | 48 | 0.013111 | 0.019114 | increase |
| XBB1006 | 0.044592 | 0.012471 | 72 | 39 | 0.000677 | 0.001261 | increase |
| Lachnospiraceae.UCG.001 | 0.0485 | 0.013251 | 72 | 53 | 0.000485 | 0.000978 | increase |
| Frisingicoccus | 0.048572 | 0.010704 | 72 | 46 | 2.30E-05 | 5.92E-05 | increase |
| Family.XIII.UCG.001 | 0.049238 | 0.010022 | 72 | 67 | 6.70E-06 | 1.93E-05 | increase |
| p.1088.a5.gut.group | 0.049721 | 0.013023 | 72 | 65 | 0.000288 | 0.000611 | increase |
| UCG.005 | 0.05193 | 0.010587 | 72 | 71 | 5.85E-06 | 1.73E-05 | increase |
| Family.XIII.AD3011.group | 0.052185 | 0.008586 | 72 | 71 | 7.83E-08 | 3.38E-07 | increase |
| X.Anaerorhabdus..furcosa.group | 0.053468 | 0.008515 | 72 | 67 | 3.54E-08 | 1.59E-07 | increase |
| Lachnospiraceae.NK4B4.group | 0.054871 | 0.009684 | 72 | 49 | 3.01E-07 | 1.07E-06 | increase |
| Clostridium.sensu.stricto.1 | 0.057217 | 0.008316 | 72 | 72 | 3.23E-09 | 2.30E-08 | increase |
| X.Eubacterium..brachy.group | 0.057283 | 0.009688 | 72 | 53 | 1.49E-07 | 5.82E-07 | increase |
| Candidatus.Saccharimonas | 0.057437 | 0.014146 | 72 | 55 | 0.000126 | 0.000288 | increase |
| Quinella | 0.059511 | 0.009056 | 72 | 31 | 1.11E-08 | 5.79E-08 | increase |
| dgA.11.gut.group | 0.059895 | 0.00926 | 72 | 70 | 1.15E-08 | 5.79E-08 | increase |
| Lachnospiraceae.UCG.007 | 0.061499 | 0.007592 | 72 | 40 | 1.19E-11 | 1.31E-10 | increase |
| Candidatus.Soleaferrea | 0.066799 | 0.010718 | 72 | 67 | 3.03E-08 | 1.41E-07 | increase |
| Lachnospiraceae.AC2044.group | 0.071135 | 0.012198 | 72 | 63 | 2.05E-07 | 7.51E-07 | increase |
| Bacteroides | 0.075565 | 0.020057 | 72 | 65 | 0.00034 | 0.00071 | increase |
| Prevotellaceae.UCG.001 | 0.076137 | 0.02126 | 72 | 61 | 0.000627 | 0.001205 | increase |
| Terrisporobacter | 0.077184 | 0.011642 | 72 | 70 | 8.80E-09 | 4.84E-08 | increase |
| Treponema | 0.089811 | 0.012395 | 72 | 71 | 4.45E-10 | 3.84E-09 | increase |
| Anaeroplasma | 0.096057 | 0.008927 | 72 | 51 | 1.74E-16 | 5.26E-15 | increase |
| Romboutsia | 0.126062 | 0.014671 | 72 | 54 | 1.48E-12 | 1.99E-11 | increase |
| Fibrobacter | 0.13004 | 0.011243 | 72 | 61 | 6.67E-18 | 4.03E-16 | increase |
| Cellulosilyticum | 0.132838 | 0.011778 | 72 | 51 | 2.12E-17 | 8.53E-16 | increase |
| Papillibacter | 0.138037 | 0.016378 | 72 | 34 | 6.37E-12 | 7.71E-11 | increase |
| Turicibacter | 0.143535 | 0.013771 | 72 | 64 | 2.42E-15 | 5.86E-14 | increase |
| Lachnospiraceae.XPB1014.group | 0.148002 | 0.015997 | 72 | 52 | 9.15E-14 | 1.84E-12 | increase |

*Coeff*- the model coefficient value (effect size*), stderr*: the standard error from the model; *N:* the total number of samples used in the model for this association *N.not.0*: the total of number of these samples in which the feature is non-zero. *pval*: the significance of this association. *qval*: the corrected significance is computed with *p.adjust* using the BH correction method.
