# Supplementary material for: Temporal dynamics of the fecal microbiome in female pigs from early life through estrus, parturition, and weaning of the first litter of piglets
Source: Anim Microbiome. 2024 Feb 21;6:7. doi: 10.1186/s42523-024-00294-8 (PMC10882843; doi:10.1186/s42523-024-00294-8)
Supplement: Supplementary file 6 — Additional File 6. Figure S4: Relative abundance of fungi at A) Phylum and B) Family levels, and C) distance to centroid (beta dispersion of overall fecal fungi composition at genus level) for each age point. [file 42523_2024_294_MOESM6_ESM.docx]

**
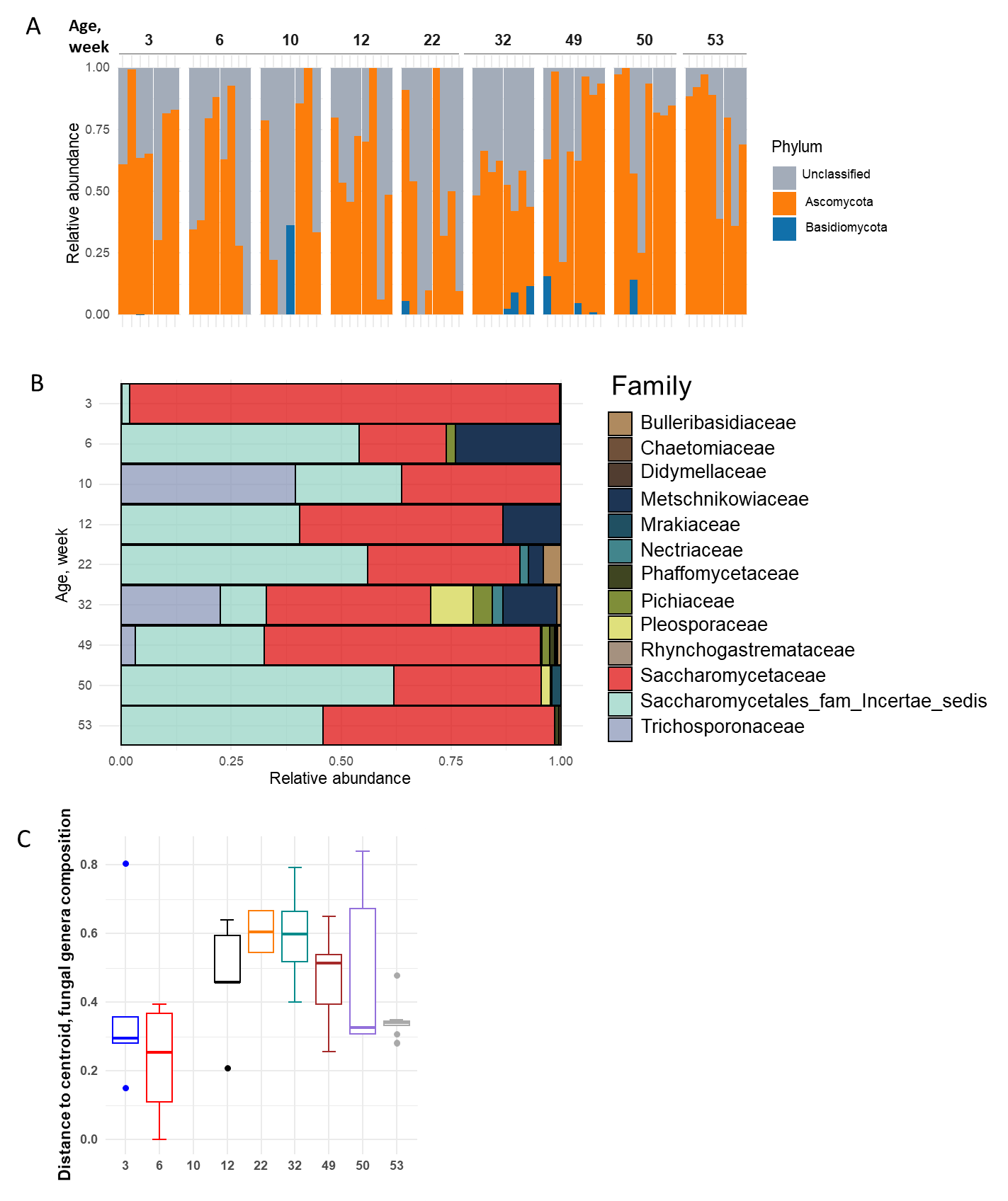
**

**Additional File 6. FigS4.** Relative abundance of fungi at A) phylum and B) family levels, and C) distance to centroid (beta dispersion of overall fecal fungi composition at genus level) for each age group.
