# Supplementary material for: Temporal dynamics of the fecal microbiome in female pigs from early life through estrus, parturition, and weaning of the first litter of piglets
Source: Anim Microbiome. 2024 Feb 21;6:7. doi: 10.1186/s42523-024-00294-8 (PMC10882843; doi:10.1186/s42523-024-00294-8)
Supplement: Supplementary file 7 — Additional File 7. Figure S5: Mean changes in (A) nonstandardized log10 tet(A), (B) nonstandardized log10 blaCTX-M, (C) standardized (to 16S rRNA) log10 tet(A), and (D) standardized (to 16S rRNA) log10 blaCTX-M log10 gene copy numbers per gram wet feces over time (pig age in weeks), and (E) Total log10 16S rRNA gene copies per gram wet feces over time (pig age in weeks). [file 42523_2024_294_MOESM7_ESM.docx]

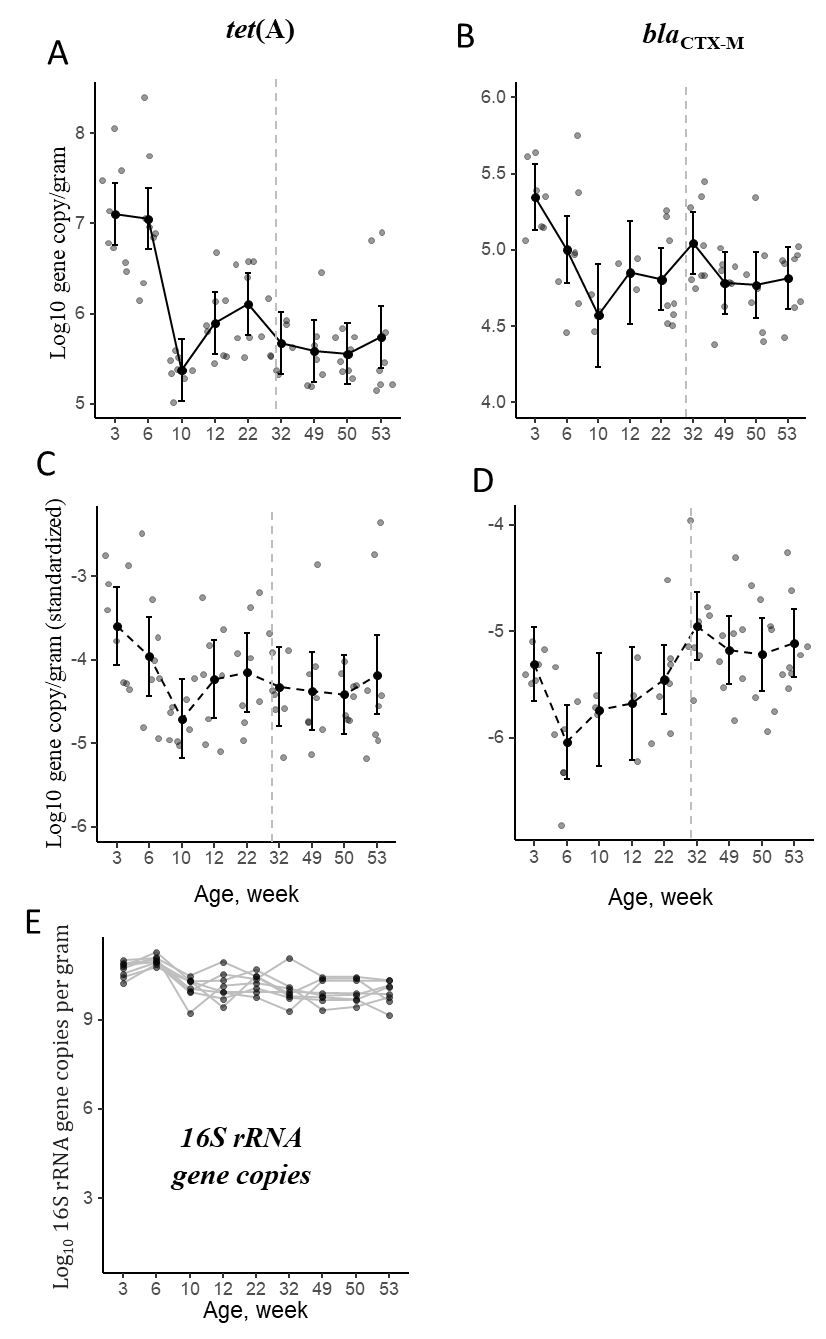


**Additional File 7. FigS5.** Mean changes in (A) nonstandardized log10 *tet*(A), (B) nonstandardized log10 *bla*_CTX-M_, (C) standardized (to 16S rRNA) log10 *tet*(A), and (D) standardized (to 16S rRNA) log10 *bla*_CTX-M_ log_10_ gene copy numbers per gram wet feces over time (pig age in weeks), and (E) Total log10 16S rRNA gene copies per gram wet feces over time (pig age in weeks)
